# Genetic Variability in Molecular Pathways Implicated in Alzheimer's Disease: A Comprehensive Review

**DOI:** 10.3389/fnagi.2021.646901

**Published:** 2021-03-18

**Authors:** David Vogrinc, Katja Goričar, Vita Dolžan

**Affiliations:** Pharmacogenetics Laboratory, Institute of Biochemistry and Molecular Genetics, Faculty of Medicine, University of Ljubljana, Ljubljana, Slovenia

**Keywords:** Alzheimer's disease, genetics, biomarker, molecular pathways, gene ontology

## Abstract

Alzheimer's disease (AD) is a complex neurodegenerative disease, affecting a significant part of the population. The majority of AD cases occur in the elderly with a typical age of onset of the disease above 65 years. AD presents a major burden for the healthcare system and since population is rapidly aging, the burden of the disease will increase in the future. However, no effective drug treatment for a full-blown disease has been developed to date. The genetic background of AD is extensively studied; numerous genome-wide association studies (GWAS) identified significant genes associated with increased risk of AD development. This review summarizes more than 100 risk loci. Many of them may serve as biomarkers of AD progression, even in the preclinical stage of the disease. Furthermore, we used GWAS data to identify key pathways of AD pathogenesis: cellular processes, metabolic processes, biological regulation, localization, transport, regulation of cellular processes, and neurological system processes. Gene clustering into molecular pathways can provide background for identification of novel molecular targets and may support the development of tailored and personalized treatment of AD.

## Introduction

Alzheimer's disease (AD) is a progressive neurodegenerative disorder, affecting the cerebral cortex and hippocampus in human brain (Masters et al., 2015). The mechanisms of disease pathogenesis are still not entirely elucidated (Kocahan and Doğan, 2017). The accumulation of amyloid-β (Aβ) in form of insoluble plaques and aggregation of protein tau in neuronal neurofibrillary tangles (NFT) are considered as two important hallmarks of AD (Masters et al., 2015).

AD is the most common neurodegenerative brain disease and a significant part of worldwide population is affected. AD, as the leading cause of dementia, contributes to 60–65% of all cognitive decline cases (Rizzi et al., 2014). Reports suggest that roughly 47 million people suffered from dementia in 2015 (Prince, 2015). The mean incidence of AD is estimated to 1–3%, with a prevalence of 10–30% in population above 65 years of age (Kawas et al., 2000; 2020 Alzheimer's disease facts figures, 2020). As population is aging, the prevalence will increase, making dementia one of the most important health issues in the future. Projections suggest that more than 13 million people will suffer from AD in the United States alone and 11.8% of all people globally will be affected by the 2050 (Brookmeyer et al., 2007; Hebert et al., 2013).

A small proportion of AD cases show familial, highly inheritable form of AD, contributing to <1% of AD. Early age of onset is associated with this type of AD, that is also known as dominant inherited Alzheimer's disease (Masters et al., 2015). Furthermore, mutations in three common genes—amyloid precursor protein (*APP*), presenilin-1 (*PSEN1*), and presenilin-2 (*PSEN2*) are associated with early-onset AD (EOAD), developing in fourth or fifth decade of life (Mayeux and Stern, 2012; Naj and Schellenberg, 2017). However, not all EOAD cases can be explained with these mutations. Late-onset AD (LOAD) cases comprise the vast majority of all AD patients (>90%), with the typical age of onset above 65 years (Bekris et al., 2010). Complex genetic and environmental interactions have been associated with risk for sporadic LOAD (Miyashita et al., 2013; Masters et al., 2015). Studies suggest LOAD is not as strongly linked to familial background as EOAD, but genetic factors can contribute importantly to AD risk even late in life (Pedersen et al., 2004; Gatz et al., 2005). Contrary to EOAD, there are no highly penetrant mutations in a set of known genes; instead multiple low penetrance genetic variants can confer risk for LOAD (Naj and Schellenberg, 2017).

Although AD generally manifests in older population, first changes in biomarkers levels, such as Aβ_42_ and phosphorylated tau (ptau_181_) can be observed already 15–20 years prior to the onset of the clinical symptoms (Blennow et al., 2010; Efthymiou and Goate, 2017). Furthermore, functional and molecular imaging of brain with single-photon emission computed tomography and positron emission tomography (PET) provides valuable early information about the underlying pathological processes such as glucose metabolism, accumulation of tau and Aβ or neuroinflammation (Valotassiou et al., 2018). Biomarkers are usually used to inform and support the diagnostic of the disease when cognitive decline has already become apparent (Efthymiou and Goate, 2017). Using biomarkers for improved diagnostic in non-demented individuals could contribute to better understanding of neurodegenerative changes late in life and support development and implementation of novel therapeutic approaches.

Some asymptomatic changes that precede typical AD cognitive symptoms can be observed in patients before the clinical diagnosis. For instance, increased biomarkers levels in adults without symptoms of cognitive impairment are typical for preclinical AD, whereas the earliest symptomatic stage when cognitive symptoms are present, but not reaching the threshold for AD dementia diagnosis, is known as prodromal AD (Dubois et al., 2016). Another clinical stage associated with AD is mild cognitive impairment (MCI). MCI is a clinical stage of progressive cognitive impairment exceeding the expected cognitive decline for age and education status (Petersen et al., 1999; Lee et al., 2017). Since around 50% of MCI patients develop AD in 5 years from diagnosis, MCI is often considered as an intermediate stage between normal aging and AD (Petersen et al., 1999). Adults with diagnosed MCI show milder cognitive decline and higher degree of independence in functional status than patients with AD (Langa and Levine, 2014). There are several studies trying to detect or predict the conversion from MCI to AD (Davatzikos et al., 2011; Sun et al., 2017; Hojjati et al., 2018). Since therapeutic interventions are more efficient during the MCI or in early stage of AD, sensitive and reliable methods for identification of cognitive decline should be used in clinical practice (Olazaran et al., 2004; Cummings et al., 2007; Buschert et al., 2011).

Common genetic polymorphisms in genes that encode proteins involved in different biological pathways implicated in the pathogenesis of LOAD could influence its development and progression. This review summarizes the latest knowledge on genetics and genomics of AD susceptibility, compiled by GWASs and their meta-analyses. In addition we have performed gene clustering of the genomic loci and molecular pathways in development and progression of MCI and AD with the aim of facilitating identification of novel biomarkers or treatment targets.

## Methods

**Figure 1:**
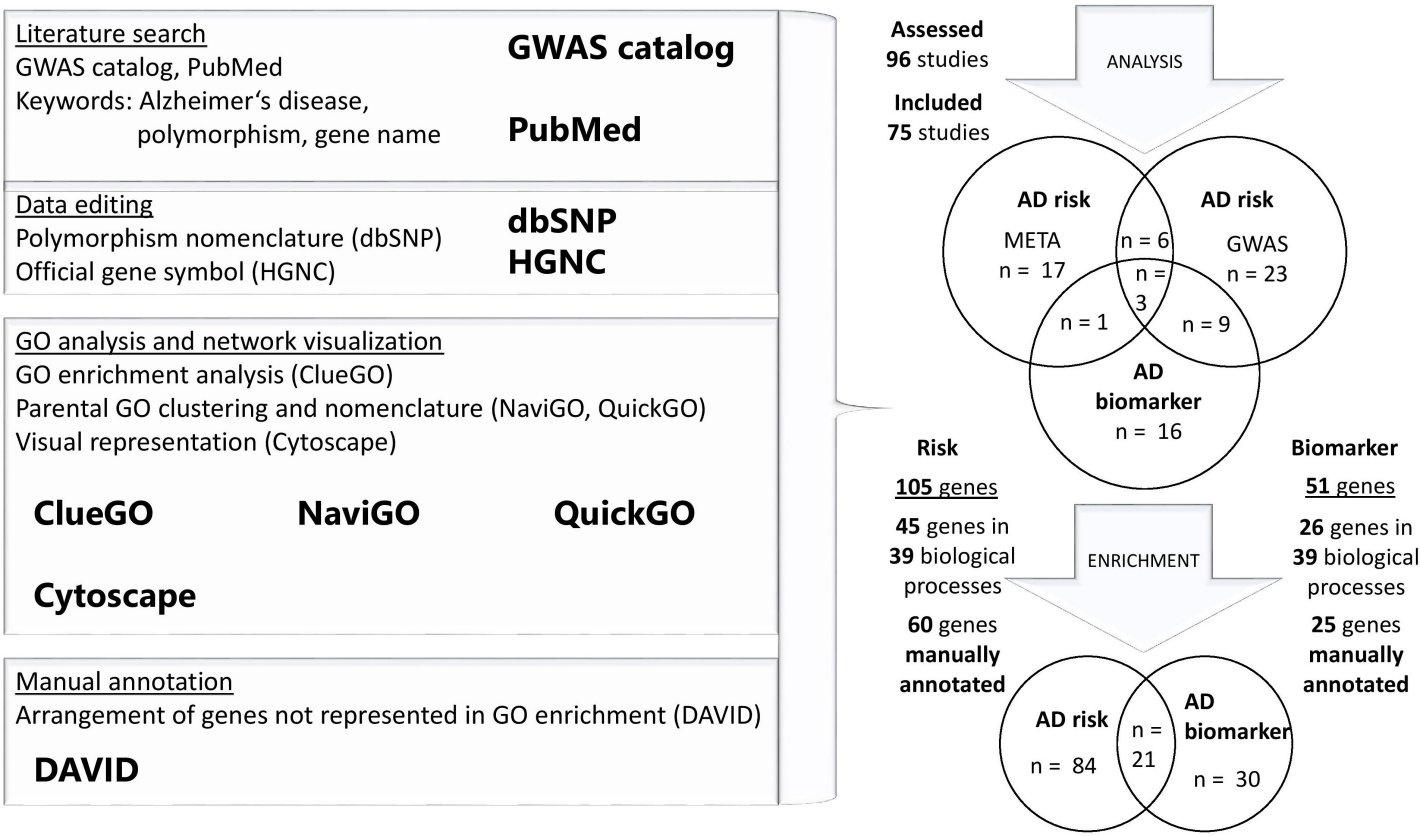
Flowchart of the study design and GO analysis. Literature search of GWAS and meta-analyses was performed in “GWAS catalog,” to obtain a list of AD-related genes. All of the studies were manually reviewed and some further literature search of identified gene loci was performed in PubMed. One hundred and five AD risk loci and 30 loci related to biomarker oscillations were used for GO enrichment analysis in two separate gene sets. Genes that were not enriched in performed GO analysis, were manually annotated to corresponding categories.

A literature search was performed in NHGRI-EBI platform “GWAS catalog,” aiming to systematically gather vast dataset of genome-wide studies and meta-analysis of complex diseases (Buniello et al., 2019). A total of 96 GWAS and meta-analyses were included in the database until the end of December 2019. For each loci, identified by GWAS, a PubMed literature search was performed with the help of the following words: “Alzheimer's disease and *gene name*” or “Alzheimer's disease and polymorphisms and *gene name*.” Novel references, assessing the risk for the disease on the genome-wide level, were included in the review. Applied exclusion criteria were expression studies, studies not implementing case-control design, studies overlapping with other diseases and studies performed on a defined set of genes—not genome-wide design (Supplementary Figure 1). In total, GWAS (*n* = 54) and meta-analyses combining multiple GWAS dataset (*n* = 21) in AD risk evaluation were included (Supplementary Tables 1, 2). Nine studies combined GWAS and meta-analysis approach in identifying AD risk loci. Studies evaluating the association of GWAS and meta-analysis dataset with disease biomarkers (*n* = 16) were analyzed separately (Figure 1). Multiple studies (*n* = 13) combined identified genotype alterations in GWAS and meta-analyses with changes in AD-related biomarkers (Figure 1). A total of 105 AD risk loci were identified with additional 30 loci related to biomarker oscillations.

For the obtained gene loci, Gene Ontology (GO) enrichment analysis was performed, using Cytoscape plug-in ClueGO (Figure 1). This tool enables to find statistically overrepresented GO pathways in a set of genes and their visual representation in a functional network (Bindea et al., 2009). We focused on GO—biological process only. Analysis for AD risk and biomarker set of genes was performed separately (Supplementary Tables 3–6). Next, list of GO overrepresented pathways was visualized with NaviGO analytic tool, to find common GO parental pathways (Wei et al., 2017). Terms were manually curated with QuickGO web browser (Binns et al., 2009). Genes that did not reach significant threshold in GO analysis, were manually annotated in one of the identified categories, using DAVID functional annotation tool (Figure 1) (Huang et al., 2009).

## Genes and Molecular Pathways Implicated in MCI and AD Risk

**Figure 2:**
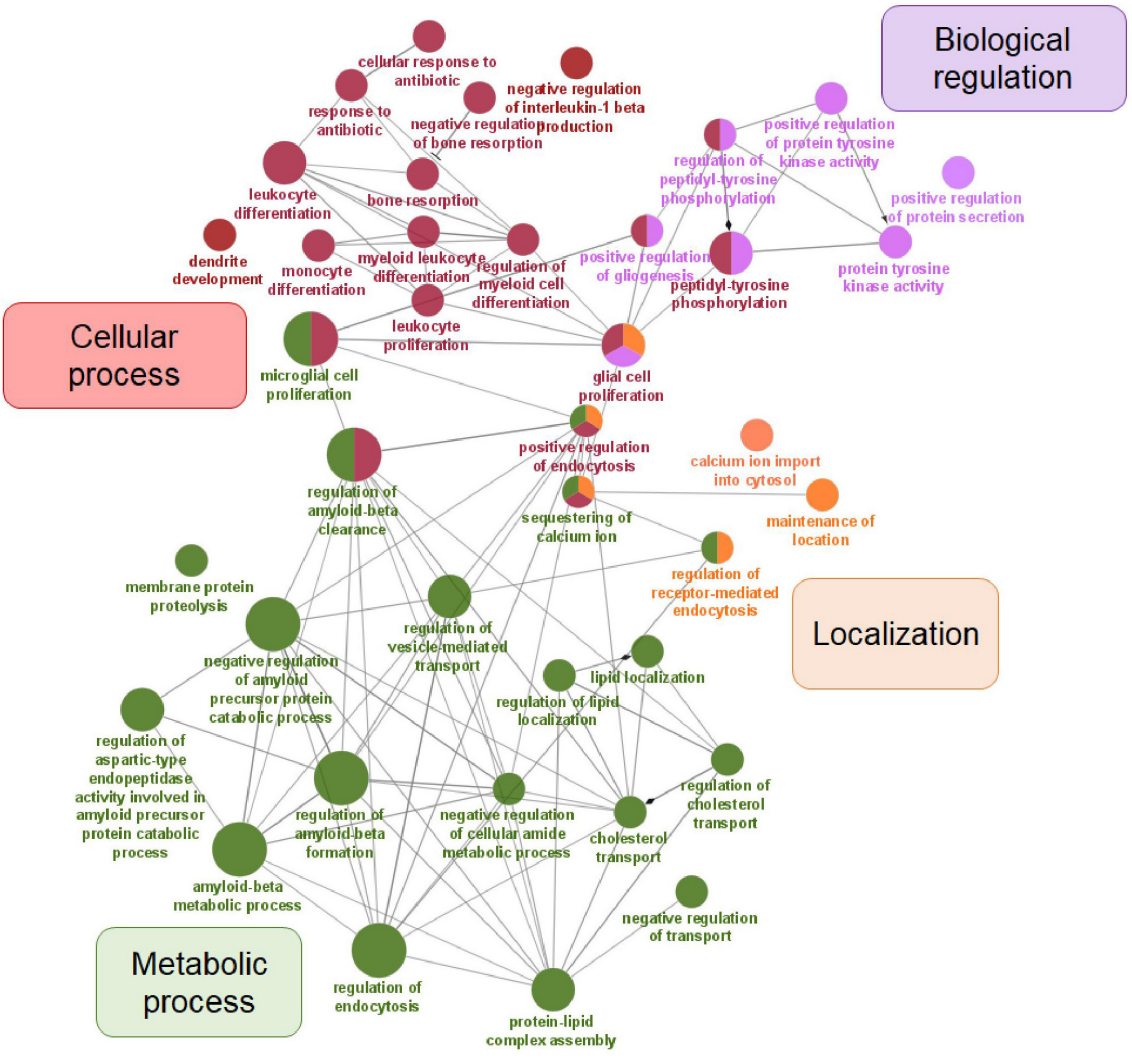
Visualization of GO analysis in AD risk gene set. Genes associated with AD risk were stratified according to GO – biological process. They are clustered in four parental categories and represented with specific color of the node. Biological processes that can be assigned to multiple parental categories, are represented with multiple color-pie chart.

In our dataset of genes related to AD risk, we observed significant enrichment for four major GO biological process categories: cellular process, metabolic process, biological regulation, and localization (Figure 2).

### Metabolic Processes

Since accumulation of insoluble proteins like Aβ is one of the hallmarks in neuropathology of AD, different metabolic processes are involved in their processing. Aβ is proteolytic product of APP cleavage by enzymes of the γ- and β-secretase (BACE) family that includes PS1 and PS2 (Masters et al., 2015). Studies of inherited form of EOAD suggest that mutations in *APP, PSEN1*, and *PSEN2* genes result in overproduction of the hydrophobic Aβ_40_ and Aβ_42_ peptides, leading to aggregation and formation of insoluble plaques (Golde et al., 2000; Pimplikar, 2009; Masters et al., 2015). Normally, Aβ plaques are being degraded and cleared in processes driven by glial cells (Ries and Sastre, 2016). Insufficient clearance, due to the excessive aggregation of plaques, can affect surrounding synapses (Masters et al., 2015).

Several lines of evidence support the genetic basis of amyloid cascade hypothesis. Firstly, known mutations in *APP, PSEN1*, and *PSEN2* genes associated with familial AD or EOAD affect the generation or aggregation propensity of Aβ (Heppner et al., 2015). Secondly, the *APP* gene is located on 21th chromosome and patients with Down's syndrome (the trisomy of the 21 chromosome) have increased risk for early development of memory impairment (García-Alba et al., 2019). Thirdly, apolipoprotein E (*APOE*) E4 allele (*APOE4*), which is associated with more extensive Aβ deposition is considered a major risk factor for LOAD (Amemori et al., 2015). It is estimated that 40–65% of AD patients have at least one copy of this allele (Namba and Ikeda, 1991; Olgiati et al., 2011). However, no successful therapies targeting amyloid accumulation have been implemented to date, suggesting the importance of other pathways that are also disrupted in AD (Efthymiou and Goate, 2017).

Genes and key SNPs included in metabolic processes, associated with AD risk in GWAS and meta-analyses, are summarized in Table 1.

**Table 1:** Genes in metabolic processes influencing AD risk.

**Gene locus**	**SNP**	**Significance for AD risk**	**OR (95 % CI)**	**Study**
*ABCA7*	rs3752246 G>C rs3764650 T>G rs4147929 A>G rs115550680 C>G rs111278892 C>G	3.1 × 10^−16^ 4.5 × 10^−17^ 1.1 × 10^−15^ 2.21 × 10^−9^ 7.93 × 10^−11^	1.15 (1.11–1.18) 1.23 (1.18–1.30) 1.15 (1.11–1.19) 1.79 (1.47–2.12)	Kunkle et al., 2019 Hollingworth et al., 2011 Lambert et al., 2013b Reitz et al., 2013 Jansen et al., 2019
*ADAM10*	rs593742 A>G rs442495 T>C	6.20 × 10^−11^ 6.8 × 10^−9^ 1.31 × 10^−9^	1.06 (1.04–1.07) 0.93 (0.91–0.95)	Marioni et al., 2018 Kunkle et al., 2019 Jansen et al., 2019
*APH1B*	rs117618017 C>T	3.35 × 10^−8^		Jansen et al., 2019
*APOC1*	rs4420638 A>G	5.30 × 10^−34^ 5.30 × 10^−14^ 1.97 × 10^−14^ 5.62 × 10^−27^ 4.58 × 10^−37^	4.01 3.2 (2.68–3.9)	Coon et al., 2007 Bertram et al., 2008 Li et al., 2017 De Jager et al., 2012 Webster et al., 2010
*APOE*	rs429358 T>C rs405509 T>G	3.66 × 10^−11^ 1.2 × 10^−881^ 4.9 × 10^−37^	3.32 (3.20–3.45) 0.70 (0.66–0.74)	Davies et al., 2014 Kunkle et al., 2019 Harold et al., 2009
*ATP8B4*	rs10519262 G>A	4.48 × 10^−6^	1.89 (1.46–2.45)	Li et al., 2008a
*BIN1*	rs744373 A>G rs6733839 C>T rs7561528 G>A rs4663105 A>C	1.6 × 10^−11^ 6.9 × 10^−44^ 2.1 × 10^−44^ 4.0 × 10^−14^ 3.38 × 10^−44^	1.13 (1.06–1.21) 1.22 (1.18–1.25) 1.20 (1.17–1.23) 1.17 (1.13–1.22)	Seshadri et al., 2010 Lambert et al., 2013b Kunkle et al., 2019 Naj et al., 2011 Jansen et al., 2019
*CELF1*	rs10838725 T>C	1.1 × 10^−8^	1.08 (1.05–1.11)	Lambert et al., 2013b; Hinney et al., 2014
*CRY2*	rs12805422 G>A	1.57 × 10^−13^		Zhu et al., 2019
*FRMD4A*	rs7081208 G>A rs2446581 G>A rs17314229 C>T	0.0202 2.1 × 10^−5^ 0.0494	1.06 (1.01–1.12) 1.15 (1.08–1.24) 1.09 (1.00–1.20)	Lambert et al., 2013a
*OSBPL6*	rs1347297 C>T	4.53 × 10^−8^		Herold et al., 2016
*PICALM*	rs3851179 T>C rs10792832 A>G rs561655 G>A rs867611 G>A	1.9 × 10^−8^ 3.16 × 10^−12^ 6.0 × 10^−25^ 9.3 × 10^−26^ 7.0 × 10^−11^ 2.19 × 10^−18^	0.85 (0.80–0.90) 0.87 (0.84–0.91) 0.88 (0.86–0.90 0.87 (0.85–0.89) 0.87 (0.84–0.91)	Harold et al., 2009 Seshadri et al., 2010 Kunkle et al., 2019 Lambert et al., 2013b Naj et al., 2011 Jansen et al., 2019
*RAB20*	rs56378310 A>G	1.52 × 10^−6^		Lee et al., 2017
*SLC10A2*	rs16961023 C>G	4.59 × 10^−8^	0.41 (0.27–0.55)	Mez et al., 2017
*SPPL2A*	rs59685680 T>G	9.17 × 10^−9^		Marioni et al., 2018
*VSNL1*	rs4038131 A>G	5.9 × 10^−7^	0.65	Hollingworth et al., 2012
**Manually annotated genes**				
*ADAMTS4*	rs4575098 G>A	2.05 × 10^−10^		Jansen et al., 2019
*ADARB2*	rs10903488 T>C	3.24 × 10^−6^		Lee et al., 2017
*ALPK2*	rs76726049 T>C	3.30 × 10^−8^		Jansen et al., 2019
*ATP5MC2*	rs1800634 T>C	6.0 × 10^−4^		Meda et al., 2012
*BCKDK*	rs889555 C>T	4.11 × 10^−8^	0.95 (0.94–0.97)	Marioni et al., 2018
*BDH1*	rs2484 T>C	2.83 × 10^−6^		Lee et al., 2017
*CELF2*	rs201119 T>C	1.5 × 10^−8^	4.02	Wijsman et al., 2011
*CRYL1*	rs7989332 A>C	0.006		Gusareva et al., 2014
*ECHDC3*	rs11257238 T>C	1.26 × 10^−8^		Jansen et al., 2019
*FBXL7*	rs75002042 T>A	6.19 × 10^−9^	0.61 (0.52–0.71)	Tosto et al., 2015
*GALNT7*	rs62341097 G>A	6.00 × 10^−9^	0.21 (0.08–0.57)	Beecham et al., 2014
*GLIS3*	rs514716 C>T	2.94 × 10^−8^	0.954	Deming et al., 2017
*KAT8*	rs59735493 G>A	3.98 × 10^−8^		Jansen et al., 2019
*KHDRBS2*	rs6455128 A>C	0.006		Gusareva et al., 2014
*NFIC*	rs9749589 T>A	1.5 × 10^−8^	0.76 (0.69–0.83)	Jun et al., 2017
*PCK1*	rs8192708 A>G	9.9 × 10^−5^	1.29 (1.12–1.49)	Grupe et al., 2007
*SPSB1*	rs11121365 C>G	1.92 × 10^−6^		Lee et al., 2017
*ST6GAL1*	rs3936289 T>C	4.92 × 10^−7^		Lee et al., 2017

Among all AD-related genetic risk factors, *APOE* on chromosome 19 is considered the strongest one and is also the most investigated in the literature. Two common *APOE* polymorphisms, rs429358 (p.Cys112Arg) and rs7412 (p.Arg158Cys) define polymorphic alleles *APOE2, APOE3*, and *APOE4* that encode three respective protein variants: apoE2 (Cys112, Cys158), apoE3 (Cys112, Arg158), and apoE4 (Arg112, Arg158) (Zannis et al., 1982). Substitution of one or two amino acids affects the total charge and structure of APOE, leading to alteration in binding to cellular receptors and lipoprotein particles and possibly changing the stability and rate of production and clearance (Masters et al., 2015). Among all populations*, APOE3* allele is the most frequent (50–90%), followed by *APOE4* (5–35%) and *APOE2* allele (1–5%) (Mahley and Rall, 2000). The association of *APOE4* with increased AD risk and an earlier age of onset of AD was confirmed (Corder et al., 1993; Saunders et al., 1993). One or two copies of the *APOE4* allele increases LOAD risk for 3- or 12-fold and contribute to ~50% LOAD (Ashford, 2004; Williams et al., 2020). Although *APOE4* allele is widely considered as a major genetic risk factor for AD, it is neither necessary nor sufficient for the development of the disease (Meyer et al., 1998). On the other hand, a protective effect of *APOE2* was shown (Corder et al., 1994). GWAS studies confirmed *APOE* rs429358 was associated with increased AD risk, while rs7412 was associated with decreased AD risk (Bertram et al., 2008; Shen et al., 2010; Beecham et al., 2014; Davies et al., 2014). Furthermore, *APOE* rs429358 showed increased risk for AD, while a protective role of *APOE* rs405509 was reported (Harold et al., 2009; Kunkle et al., 2019). Role of APOE in catabolism of triglyceride-rich lipoproteins is well-studied (Masters et al., 2015). APOE regulates their metabolism through binding to ApoE receptors, directing the transport, delivery, and distribution of lipoproteins (Mahley, 1988; Mahley and Rall, 2000). Discovery of APOE immunoreactivity in Aβ deposits and NFT, hallmarks of AD pathology, was an important research milestone in AD (Namba and Ikeda, 1991).

Besides *APOE*, a lot of other LOAD susceptibility loci involved in different metabolic processes have been reported to date (Table 1). Several genes play an important role in APP and tau processing, vesicle mediated transport or endocytosis. Multiple single nucleotide polymorphisms within and near phosphatidylinositol binding clathrin assembly protein (*PICALM)* gene were associated with AD. *PICALM* rs3851179 was associated with decreased AD risk (Seshadri et al., 2010; Kunkle et al., 2019). *PICALM* rs561655 showed decreased risk for LOAD and was subsequently associated with earlier age-of-onset of the disease (Naj et al., 2011, 2014). International Genomics of Alzheimer's disease project (IGAP) demonstrated an increased risk of AD associated with *PICALM* rs10792832 (Lambert et al., 2013b). Another polymorphism, *PICALM* rs867611, was confirmed as AD-related (Jansen et al., 2019). PICALM is an accessory protein in the endocytic pathway. It binds to clathrin and its adaptor proteins. Clathrin-mediated endocytosis is necessary for γ-secretase to cleave APP and form β-amyloid (Tanzi, 2012). Rs117618017 near *APH1B*, aph-1 homolog B, gamma-secretase subunit, coding for anterior pharynx defective-1 protein, another crucial part of γ-secretase complex important in APP cleaving, was also associated with AD risk (Acx et al., 2017; Jansen et al., 2019). *BIN1* (bridging integrator 1) rs744373 SNP was associated with risk for LOAD (Seshadri et al., 2010). Naj et al. confirmed association of *BIN1* rs7561528 with LOAD, while IGAP showed positive association for rs6733839 (Naj et al., 2011; Lambert et al., 2013b). Moreover, *BIN1* rs6733839 was also associated with increased AD risk (Kunkle et al., 2019). Another *BIN1* AD-related polymorphism was rs4663105 (Broce et al., 2019). BIN1 is a widely expressed adaptor protein that is part of the Bin1/amphiphysin/RVS167 (BAR) family. BIN1 functions in clathrin-mediated endocytosis and endocytic recycling (Wigge et al., 1997). It is also known as a tumor suppressor gene (Rosenthal and Kamboh, 2014). *ADAM10* rs593742 was identified as a novel AD risk locus (Marioni et al., 2018). The protective function was observed in additional study (Kunkle et al., 2019). *ADAM10* rs442495 was also associated with AD (Jansen et al., 2019). ADAM10 is as a member of ADAM family involved in the cleavage of APP in thereby influencing deposition of amyloid beta (Suh et al., 2013). Recent evidence indicated primary α-secretase function of ADAM10 in mouse models (Postina et al., 2004; Jorissen et al., 2010; Kuhn et al., 2010).

Various AD risk genes were associated with lipid metabolism. *APOC1* rs4420638 was a strongly associated risk factor for AD (Coon et al., 2007). This association was confirmed in other studies (Webster et al., 2010; De Jager et al., 2012). APOC1 is involved in lipoprotein metabolism, but is interfering with fatty acids and reducing their intracellular esterification (Westerterp et al., 2007). Two *ABCA7* SNP were associated with risk for LOAD (Hollingworth et al., 2011). Rs3752246 is the only coding non-synonymous missense SNP that may alter the function of ABCA7 protein in AD, while rs3764650 minor allele confers increased risk (Hollingworth et al., 2011; Pahnke et al., 2014; Kunkle et al., 2019). Another SNP, *ABCA7* rs4147929, was associated with increased LOAD risk (Lambert et al., 2013b). A strong association of *ABCA7* rs115550680 with increased LOAD risk was shown (Reitz et al., 2013). Furthermore, *ABCA7* rs111278892 was recently associated with AD (Jansen et al., 2019). *ABCA7* encodes an ATP-binding cassette transporter A7, which belongs to the A subfamily of ABC transporters (Hollingworth et al., 2011; Steinberg et al., 2015). Other than its role in cholesterol metabolism, recent data from mouse models suggest its role in the regulation of phagocytosis (Steinberg et al., 2015). It modulates the phagocytosis of apoptotic cells by macrophages mediated through the complement component C1q and it also participates in macrophage uptake of Aβ (Hollingworth et al., 2011; Rosenthal and Kamboh, 2014). *ABCA7* is highly expressed in hippocampal CA1 neurons and microglia (Hollingworth et al., 2011; Rosenthal and Kamboh, 2014). A reduction in *ABCA7* expression or loss of function could increase amyloid production and may contribute to AD susceptibility (Satoh et al., 2015). *SLC10A2* rs16961023 showed a protective association with LOAD (Mez et al., 2017). Na^+/^bile acid cotransporter, encoded by *SLC10A2*, is a mediator in initial bile acid adsorption and is important for cholesterol homeostasis (Love et al., 2001). *OSBPL6* rs1347297 was associated with LOAD (Herold et al., 2016). *OSBPL6* is coding for oxysterol binding protein-like-6 receptor (Assou et al., 2013). Oxysterols are oxidized form of cholesterol that are able to cross the blood-brain-barrier (Testa et al., 2018). This process prevents excessive cholesterol accumulation in brain and may have an important role in AD pathogenesis.

The communication between different regions of the cell is mediated through dynamic networks of signaling cascades (Horbinski and Chu, 2005). This process is driven by enzymes like signaling kinases that alter the expression, activity or localization of proteins through phosphorylation mechanisms (Lash and Cummings, 2010). *SPPL2A* rs59685680 was associated with AD (Marioni et al., 2018). Signal peptide SPPL2A is part of aspartic intramembrane proteases, which cleave type II transmembrane proteins (Zhang et al., 2017). Interaction with immune system components, such as TNF, were previously reported (Friedmann et al., 2006). Three polymorphisms in *FRMD4A*—rs7081208, rs2446581, rs17314229—were associated with increased AD risk (Lambert et al., 2013a). FRMD4A is involved in Par protein binding and regulates epithelial cell polarity through cytohesins (Ikenouchi and Umeda, 2010). Par-related signaling pathway plays a crucial role in neuronal polarization (Insolera et al., 2011). A protective *VSNL1* rs4038131 association with AD and psychosis was reported (Hollingworth et al., 2012). Calcium modulated VSNL1 utilizes a calcium-myristoyl switch phosphorylation, translocating the VSNL1 to cell membrane for induction of numerous cell signaling pathways (Braunewell and Szanto, 2009).

Several genes were associated with mRNA processing and transcriptional regulation. Rs10838725 in *CELF1* region was associated with increased risk for AD in IGAP (Lambert et al., 2013b). Association with both AD and obesity was shown for *CELF1* rs10838725 (Hinney et al., 2014). *CELF1*, also called *CUG-BP*, is a member of a family of proteins involved in the regulation of pre-mRNA alternative splicing (Gallo and Spickett, 2010). *CRY2* rs12805422 was associated with AD and fasting glucose (Zhu et al., 2019). Flavin adenine dinucleotide-binding protein, encoded by *CRY2* gene is important transcriptional repressor of circadian clock (Kriebs et al., 2017).

Rs10519262 near *ATP8B4* was proposed as novel risk locus in AD (Li et al., 2008b). Implicated in energy metabolism, ATP8B4 is part of P4-ATPase flippase complex, potentially involved in ATP biosynthesis and phospholipid transport (Gao et al., 2016). *RAB20* rs56378310 was linked to MCI-AD conversion (Lee et al., 2017). *RAB20* is a member of *GTPase* family, involved in apical endocytosis, that negatively regulates neurite outgrowth (Oguchi et al., 2018).

Among all AD risk loci, obtained from GWAS and meta-analyses that were not enriched in GO analysis, additional 18 were manually annotated to a corresponding metabolic process and are also summarized in Table 1. *ADAMTS4* rs4575098 was associated with AD (Jansen et al., 2019). A primary α-secretase function in APP processing was shown for ADAMTS4, Zn^2+^ metalloprotease with proteoglycan cleavage activity (Apte, 2009; Walter et al., 2019). Recently, *ECHDC3* rs11257238 was associated with AD (Jansen et al., 2019). ECHDC3 (enoyl-CoA hydratase domain-containing protein 3) is a mitochondrial protein, important in fatty acid biosynthesis and possible insulin sensing mediator (Sharma et al., 2019). *BDH1* rs2484 showed genome-wide significant association with conversion of MCI to AD (Lee et al., 2017). BDH1 (3-hydroxybutyrate dehydrogenase 1) is important as the initiator of β-hydroxybutyrate catabolism (Wang et al., 2019a). *BCKDK* rs889555 was associated with decreased AD risk (Marioni et al., 2018). BCKDK is a kinase, phosphorylating the enzyme complex of branched amino acid metabolism (Cook et al., 1984; Zigler et al., 2016). *PCK1* rs8192708 was identified as AD risk allele (Grupe et al., 2007). PCK1—phosphoenolpyruvate carboxykinase 1—is a key enzyme in gluconeogenesis (Xia et al., 2010). It catalyzes decarboxylation and phosphorylation of oxaloacetate to phosphoenolpyruvate. *CRYL1* rs7989332 interaction with another gene (*KHDRBS2*) was associated with AD (Gusareva et al., 2014). Crystallin, lambda 1 protein (CRYL1) is more known as a structural protein in lens, however it is also involved in dehydrogenation of L-gulonate in the uronate cycle, alternative pathway to metabolism of glucose (Huang et al., 2017b). *ATP5MC2* rs1800634 was associated with LOAD (Meda et al., 2012). A subunit of mitochondrial ATP synthase, important for synthesis of ATP, is encoded by *ATP5MC2* (Chen et al., 2006).

*ADARB2* rs10903488 was associated with LOAD in MCI conversion patients (Lee et al., 2017). *ADARB2* encodes a member of the double-stranded RNA adenosine deaminase family, important RNA-editing enzymes (Gentilini et al., 2017). *CELF2* rs201119 was associated with AD driven neurodegeneration in *APOE4* homozygotes (Wijsman et al., 2011). Besides *CELF1*, another member of CELF family is *CELF2*, implicated in several post-transcriptional events (Gallo and Spickett, 2010). *KHDRBS2* rs6455128 interaction with *CRYL1* was associated with AD (Gusareva et al., 2014). *KHDRBS2* is involved in RNA splicing (Malouf et al., 2014). Interaction of rs9749589 with *APOE4* status suggested *NFIC* as a novel protective locus in AD susceptibility (Jun et al., 2017). Transcriptional regulator *NFIC* is a member of Nuclear Factor-I (NF-I) family (Gronostajski, 2000). *KAT8* rs59735493 showed a genome-wide significant association with AD (Jansen et al., 2019). KAT8 is histone acetyltransferase, part of *MSL* complex involved in acetylation of nucleosomal histone H4 (Smith et al., 2006; Yuan et al., 2012). An AD protective function of *GALNT7* rs62341097 was observed (Beecham et al., 2014). GALNT is a member of N-acetylgalactosaminyltransferases, known for oncogenic role in cancer development (Hussain et al., 2016). It is involved in mucin-type O-glycosylation, post-translational modification, that stimulates intensive proliferation and metastasis of neoplastic cells (Kudryavtseva et al., 2019). A genome-wide association with MCI to AD conversion was observed in rs3936289 in the *STG6AL1* region (Lee et al., 2017). ST6GAL1 is also involved in protein glycosylation. Interactions with BACE1 were investigated and an effect on APP secretion was shown (Kitazume et al., 2001; Nakagawa et al., 2006). Through this mode of action, BACE1 is also directly linked to synaptic function (Das and Yan, 2017). *FBXL7* rs75002042 was associated with decreased LOAD risk (Tosto et al., 2015). FBXL7 is one of the F-box proteins, important subunits of E3 ubiquitin protein ligases, enzymes involved in phosphorylation-dependent ubiquitination of proteins (Rodrigues-Campos and Thompson, 2014). *SPSB1* rs11121365 was associated with AD (Lee et al., 2017). SPSB1 is another regulator of ubiquitination and proteasomal degradation of NO synthase, important in AD (Nishiya et al., 2011). *ALPK2* rs76726049 was reported as novel AD risk locus (Jansen et al., 2019). Protein alpha-kinase 2, encoded by *ALPK2* is a serine/threonine kinase, previously associated with leukemia progression (Smirnikhina et al., 2016). *GLIS3* rs514716 protective function in AD was reported (Deming et al., 2017). Involved in gene transcription, GLIS3 is a component of Krüppel-like zinc finger transcriptional regulators (Calderari et al., 2018). Through Glis3-binding sites (G3BS), target gene transcription is regulated (Kim et al., 2003).

### Cellular Processes

Genes from different levels of cellular process are also highly enriched in AD pathology. Comprehensive list of genes and key SNPs, involved in cellular processes, associated with AD risk in GWAS and their meta-analyses, are presented in Table 2.

**Table 2:** Genes in cellular processes influencing AD risk.

**Gene locus**	**SNP**	**Significance for AD risk**	**OR (95 % CI)**	**Study**
*CLDN18*	rs16847609 G>A	5.3 × 10^−7^	1.19 (1.11–1.28)	Jun et al., 2016
*CLU*	rs11136000 T>C rs1532278 T>C rs9331896 C>T rs2279590 T>C rs9331888 C>G rs4236673A>G	1.4 × 10^−9^ 7.5 × 10^−9^ 1.62 × 10^−16^ 1.9 × 10^−8^ 2.8 × 10^−25^ 4.6 × 10^−24^ 8.9 × 10^−9^ 9.4 × 10^−8^ 2.61 × 10^−19^	0.84 (0.79–0.89) 0.86 (0.81–0.90) 0.85 (0.82–0.88) 0.89 (0.85–0.92) 0.86 (0.84–0.89) 0.88 (0.85–0.90) 0.86 (0.82–0.91) 1.16 (1.10–1.23)	Harold et al., 2009 Lambert et al., 2009 Seshadri et al., 2010 Naj et al., 2011 Lambert et al., 2013b Kunkle et al., 2019 Lambert et al., 2009 Lambert et al., 2009 Jansen et al., 2019
*COBL*	rs112404845 A>T	3.8 × 10^−8^	0.47 (0.29–0.65)	Mez et al., 2017
*CR1*	rs6656401 A>G rs6701713 A>G rs2093760 A>G rs4844610 A>C	3.7 × 10^−9^ 5.7 × 10^−24^ 4.6 × 10^−10^ 1.10 × 10^−18^ 3.6 × 10^−24^	1.21 (1.14–1.29) 1.18 (1.14–1.22) 1.16 (1.11–1.22) 1.17 (1.13–1.21)	Lambert et al., 2009 Lambert et al., 2013b Naj et al., 2011 Jansen et al., 2019 Kunkle et al., 2019
*IL34*	rs4985556 C>A	3.67 × 10^−8^		Marioni et al., 2018
*INPP5D*	rs35349669 C>T rs10933431 G>C	3.2 × 10^−8^ 2.59 × 10^−9^ 8.92 × 10^−10^ 3.4 × 10^−9^	1.08 (1.05–1.11) 1.10 (1.01–1.20) 0.91 (0.88–0.94)	Lambert et al., 2013b Ruiz et al., 2014 Jansen et al., 2019 Kunkle et al., 2019
*MEF2C*	rs190982 G>A	3.2 × 10^−8^	0.93 (0.90–0.95)	Lambert et al., 2013b
*MINK1*	rs8070572 T>C	1.98 × 10^−7^	1.12 (1.07–1.17)	Broce et al., 2019
*MS4A2*	rs7933202 A>C	1.9 × 10^−19^	0.89 (0.87–0.92)	Kunkle et al., 2019
*PLEKHA1*	rs2421016 C>T	0.0031		Wang et al., 2017
*PTK2B*	rs28834970 T>C rs73223431 C>T	7.4 × 10^−14^ 6.3 × 10^−14^	1.10 (1.08–1.13) 1.10 (1.07–1.13)	Lambert et al., 2013b Kunkle et al., 2019
*SERPINB1*	rs316341 G>A	1.76 × 10^−8^	1.03	Deming et al., 2017
*SLC9A7*	rs1883255 G>A	0.0015		Meda et al., 2012
*SPI1*	rs1057233 G>A rs3740688 G>T	5.4 × 10^−6^ 5.4 × 10^−13^	0.93 (0.89–0.96) 0.92 (0.89–0.94)	Huang et al., 2017a Kunkle et al., 2019
*TP53INP1*	rs4734295 A>G rs6982393 T>C	0.0051 0.0121		Wang et al., 2017
*TREM2*	rs75932628 C>T rs143332484 C>T rs187370608 G>A	3.42 × 10^−10^ 2.7 × 10^−15^ 1.55 × 10^−14^ 1.45 × 10^−16^	2.92 (2.09–4.09) 2.08 (1.73–2.49) 1.67	Jonsson et al., 2013 Kunkle et al., 2019 Sims et al., 2017 Jansen et al., 2019
**Manually annotated genes**				
*ABI3*	rs616338 T>C rs28394864 G>A	4.56 × 10^−10^ 1.87 × 10^−8^	1.43	Sims et al., 2017 Jansen et al., 2019
*CDC42SE2*	rs382216 C>T	2.0 × 10^−7^	0.88 (0.83–0.93)	Jun et al., 2016
*CDK5RAP2*	rs10984186 G>A rs4837766 A>T	4.23 × 10^−7^ 0.010	1.426 0.39 (0.19–0.81)	Miron et al., 2018
*CNTNAP2*	rs802571 A>G rs114360492 C>T	1.26 × 10^−6^ 2.10 × 10^−9^	0.52 (0.40–0.68)	Hirano et al., 2015 Jansen et al., 2019
*DCHS2*	rs1466662 A>T	4.95 × 10^−7^	0.38	Kamboh et al., 2012b
*FERMT2*	rs17125944 T>C rs17125924 A>G	7.9 × 10^−9^ 6.71 × 10^−10^ 1.4 × 10^−9^	1.14 (1.09–1.19) 1.24 (1.04–1.48) 1.14 (1.09–1.18)	Lambert et al., 2013b Ruiz et al., 2014 Kunkle et al., 2019
*GOLM1*	rs10868366 G>T rs7019241 C>T	2.43 × 10^−4^ 2.92 × 10^−4^	0.55 (0.40–0.75) 0.54 (0.38–0.75)	Li et al., 2008a
*HLA-DRB1*	rs9271058 A>T rs9271192 C>A rs6931277 A>T	1.4 × 10^−11^ 2.9 × 10^−12^ 8.41 × 10^−11^	1.10 (1.07–1.13) 1.11 (1.08–1.15)	Kunkle et al., 2019 Lambert et al., 2013b Jansen et al., 2019
*MADD*	rs10501320 G>C	2.80 × 10^−16^		Zhu et al., 2019
*NECTIN2*	rs6857 C>T rs6859 A>G	<1.7 × 10^−8^ 3.48 × 10^−38^ 6.09 × 10^−14^		Seshadri et al., 2010 Beecham et al., 2014 Abraham et al., 2008
	rs41289512 C>G	6.9 × 10^−41^ 5.79 × 10^−276^	1.46 (1.38–1.54)	Harold et al., 2009 Jansen et al., 2019
*NME8*	rs2718058 A>G	4.8 × 10^−9^	0.93 (0.90–0.95)	Lambert et al., 2013b
*NYAP1*	rs12539172 T>C	9.3 × 10^−10^	0.92 (0.90–0.95)	Kunkle et al., 2019
*OR4S1*	rs1483121 G>A	6.10 × 10^−10^		Zhu et al., 2019
*PCDH11X*	rs5984894 A>G	3.9 × 10^−12^	1.30 (1.18–1.43)	Carrasquillo et al., 2009
*PDS5B*	rs192470679 T>C	1.55 × 10^−6^		Lee et al., 2017
*PFDN1*	rs11168036 T>G	7.1 × 10^−9^	1.08 (1.06–1.10)	Jun et al., 2017
*PTPRG*	rs7609954 G>T	3.98 × 10^−8^		Herold et al., 2016
*SCIMP*	rs7225151 G>A rs113260531 G>A	1.38 × 10^−8^ 9.16 × 10^−10^	1.10 (1.06–1.14)	Moreno-Grau et al., 2019 Jansen et al., 2019
*SHE*	rs4474240 A>C	5.01 × 10^−6^	3.42	Haddick et al., 2017
*SHROOM2*	rs2405940 G>T	3.0 × 10^−4^		Meda et al., 2012
*SORCS1*	rs2245123 T>C	7.0 × 10^−4^		Laumet et al., 2010
*TREML2*	rs9381040 C>T	1.55 × 10^−8^		Marioni et al., 2018
*TSPOAP1-AS1*	rs2632516 G>C rs2526378 A>G	4.4 × 10^−8^ 3.64 × 10^−9^	0.92 (0.91–0.94) 0.93	Jun et al., 2017 Witoelar et al., 2018

Numerous SNPs in clusterin (*CLU)* were linked to AD. Rs11136000 was associated with decreased risk for AD (Harold et al., 2009; Lambert et al., 2009; Seshadri et al., 2010). Furthermore, *CLU* rs1532278 and rs9331896 were associated with decreased LOAD risk (Naj et al., 2011; Lambert et al., 2013b; Kunkle et al., 2019). Two other polymorphisms in *CLU* were associated with decreased (rs2279590) or increased (rs9331888) risk for AD (Lambert et al., 2009). Another novel SNP in this region, rs4236673, was also associated with AD risk (Jansen et al., 2019). CLU is a chaperone molecule that may be involved in membrane recycling and apoptosis. It interacts with soluble form of Aβ, forming complexes that cross the blood-brain barrier (Olgiati et al., 2011). It is one of the primary chaperones for removal of Aβ from the brain (Rosenthal and Kamboh, 2014). Association of *PTK2B* rs28834970 with increased AD risk was observed in IGAP (Lambert et al., 2013b). In another study, the same effect was observed for *PTK2B* rs73223431 (Kunkle et al., 2019). PTK2B is protein-tyrosine kinase, involved in multiple cellular processes. Importance of mouse homolog in Aβ signaling and therefore a potential risk for AD was proposed (Salazar et al., 2019). *CLDN18* rs16847609 was associated with increased AD risk (Jun et al., 2016). Although not expressed in nervous tissue, claudins are protein components of epithelial and endothelial tight junctions of multiple tissues, regulating cell permeability and maintaining polarity (Luo et al., 2018). Two SNPs in *TP53INP1* (rs4734295, rs6982393) were associated with AD and type 2 diabetes (T2D), indicating potential shared molecular pathways between the diseases (Wang et al., 2017). *TP53INP1* encodes a protein, involved in apoptosis and regulating cellular-extracellular matrix adhesion and cell migration (Seux et al., 2011). Mez et al. reported *COBL* rs112404845 as a novel protective locus for AD (Mez et al., 2017). COBL, a recently discovered protein, plays a role in cellular morphogenesis by regulating cytoskeletal dynamics (Ahuja et al., 2007; Hou et al., 2015). In neurons, COBL-induced actin nucleation plays a crucial role in neuritogenesis and dendritic branching (Ahuja et al., 2007).

*SLC9A7* rs1883255 was associated with LOAD (Meda et al., 2012). *SLC9A7* encodes for (Na^+^, K^+^)/proton (H^+^) exchanger in the Golgi, important in maintenance of homeostasis (Numata and Orlowski, 2001). Polymorphism rs8070572 in *MINK1* region showed higher risk for AD (Broce et al., 2019). *MINK1* encodes a serine-threonine kinase, involved in different cell processes, including dendrite development (Yu et al., 2020). A novel significant association with both AD and T2D in *PLEKHA1* rs2421016 was observed (Wang et al., 2017). Pleckstrin homology domain-containing family A member 1 protein is another mediator in cellular signaling, encoded by *PLEKHA1* (Huang and Xiang, 2018). In *MS4A* region, *MS4A2* rs7933202 was associated with AD (Kunkle et al., 2019). *MS4A* (membrane spanning 4A) cluster encodes a family of cell surface proteins that participate in the regulation of calcium signaling (Hollingworth et al., 2011; Ma et al., 2015). Their function in neurodegeneration and AD has been discussed lately (LaFerla, 2002; Marambaud et al., 2009; Hermes et al., 2010; Seaton et al., 2011; Zündorf and Reiser, 2011).

There is growing evidence that immune system is involved in the early stages of AD pathogenesis. Immune processes may drive AD pathology independently of Aβ deposition and thus sustain increased Aβ levels (Heppner et al., 2015). Immune system processes are characterized by the activation of glial cells and release of pro-inflammatory cytokines and chemokines (Liu and Chan, 2014). Recently, a rare variant with comparable effects to those of *APOE4* was identified in *TREM2* association study (Rosenthal et al., 2015). *TREM2* rs75932628 results in the substitution of a histidine for arginine at amino acid residue 47 (p.His47Arg) and was shown to considerably increase AD risk (Jonsson et al., 2013). Interestingly, this variant was associated with a significantly younger age at symptom onset compared to individuals with no *TREM2* variants (Slattery et al., 2014). The positive association of *TREM2* rs75932628 with LOAD was also replicated (Kunkle et al., 2019). *TREM2* rs143332484 conferred greater AD risk, while *TREM2* rs187370608 was significantly associated with AD (Sims et al., 2017; Jansen et al., 2019). TREM2 is a surface receptor in the plasma membrane of brain microglia, forming an immune-signaling complex with DAP12 (Sims et al., 2017). It has an important function in innate immunity and also anti-inflammatory properties (Yaghmoor et al., 2014). It is also involved in the clearance of neural debris from CNS in phagocytosis mediated mechanism, leading to the production of reactive oxygen species (Neumann and Daly, 2013). In the human brain, TREM2 is found at high concentrations in white matter, the hippocampus and the neocortex, but at very low concentrations in the cerebellum. These regions are consistent with the distribution of pathology in AD (Yaghmoor et al., 2014). *IL34* rs4985556 was recently recognized as a susceptibility locus (Marioni et al., 2018). IL34 is a homodimeric cytokine, stimulating proliferation of monocytes and macrophages through the colony-stimulating factor 1 receptor (CSF1R) (Lin et al., 2008). The effect of IL34 on microglia in AD pathogenesis was shown (Mizuno et al., 2011). Recent GWASs have shown that *CR1* genetic variations are associated with global cognitive decline and higher burden of AD brain pathology. Association between *CR1* rs6656401 and increased AD risk was observed (Lambert et al., 2009). The effect was later confirmed in IGAP study (Lambert et al., 2013b). Naj et al. confirmed the association between *CR1* rs6701713 and LOAD risk and later showed that rs6701713 was also associated with age-at-onset (Naj et al., 2011, 2014). Additionally, *CR1* rs2093760 was associated with AD and rs4844610 with increased LOAD risk (Jansen et al., 2019; Kunkle et al., 2019). Complement component receptor (CR1) is a receptor for complement fragments C3b and C4b (Madeo and Frieri, 2013) and it regulates complement cascade *via* the inhibition of both classical and alternative pathway C3 and C5 convertases (Zhu et al., 2015). Complement inhibition can reduce the clearance of Aβ in animal models (Wyss-Coray et al., 2002). Increased LOAD risk for *INPP5D* rs35349669 was identified in IGAP and confirmed in a follow-up study (Lambert et al., 2013b; Ruiz et al., 2014). The genome-wide significant association of *INPP5D* rs10933431 was observed, suggesting protective function (Jansen et al., 2019; Kunkle et al., 2019). INPP5D (Inositol Polyphospate-5 Phosphatase) regulates cytokine signaling and inhibition of PI3K-driven oncogenic pathway. It controls degradation of IgE receptor complex together with CD2AP (Rosenthal and Kamboh, 2014).

*MEF2C* rs190982 polymorphism was associated with decreased LOAD in IGAP (Lambert et al., 2013b). MEF2C is an important transcription factor, involved in the control of inflammation in vascular endothelial cells, inhibition of leukocyte transport, regulation of NF-κB activity and expression of pro-inflammatory genes (Xu et al., 2015). *SERPINB1* rs316341 was identified as novel risk locus (Deming et al., 2017). Serpins are a family of protease inhibitors. Their potential role in inhibition of Aβ toxicity has been proposed, potentially through regulation of neutrophil infiltration in immune system (Schubert, 1997; Farley et al., 2012). The protective function of *SPI1* rs1057233 and rs3740688 in AD development was proposed (Huang et al., 2017a; Kunkle et al., 2019). *SPI1* is another important gene, involved in immune system processes. It encodes PU.1, a transcription factor essential for myeloid and B-lymphoid cell development and a major regulator of cellular communication in the immune system (Huang et al., 2017a; Broce et al., 2019).

Additional 23 of the risk loci, obtained from GWAS and meta-analyses that were not enriched in GO analysis, were manually annotated to cellular processes and are also summarized in Table 2.

Two important *CDK5RAP2* SNPs were identified; rs10984186 was associated with an increased risk of developing AD while rs4837766 with opposite effect in MCI/AD risk and conversion rate was specific for women only (Miron et al., 2018). The cyclin-dependent kinase 5 regulatory subunit–associated protein 2 (CDK5RAP2) regulates cyclin-dependent kinase 5 (*CDK5*), important for tau phosphorylation and NFT formation in CNS (Arioka et al., 1993; Patrick et al., 1999). Tau protein is another important pathophysiological hallmark of AD. Under normal conditions tau binding stabilizes microtubules in axons, however, specific pathological conditions induce tau hyperphosphorylation (Di Paolo and Kim, 2011). Besides abnormal phosphorylation, tau protein aggregation in neurons can be induced, but the mechanism is not yet known (Jouanne et al., 2017). The formation of NFT due to tau aggregation can damage the neurons. The association between NFT and progression of AD has been widely studied; concentration and distribution of tangles correlated with severity and duration of dementia (Bierer et al., 1995; Gomez-Isla et al., 1997; Giannakopoulos et al., 2003).

*PFDN1* rs11168036 was associated with increased LOAD risk (Jun et al., 2017). *PFDN1* is encoding for chaperone protein that binds specifically to cytosolic chaperonin and transfers target proteins to it (Wang et al., 2015). Rs2405940 in *SHROOM2* region was associated with LOAD (Meda et al., 2012). *SHROOM2* encodes for a SHROOM family protein, an actin binding protein (Dietz et al., 2006). *NME8* rs2718058 was reported as a protective locus for AD (Lambert et al., 2013b). NME/NM23 family member 8 (NME8), is involved in numerous physiological and pathological processes, including cellular differentiation (Desvignes et al., 2010). A role of NME8 in the cytoskeletal function, axonal transport and antioxidant action has also been discussed (Liu et al., 2016). *CDC42SE2* rs382216 showed decreased risk for AD (Jun et al., 2016). CDC42SE2 is another potential actin cytoskeleton modulator, acting downstream of CDC42 (Pirone et al., 2000). Association of *FERMT2* rs17125944 with increased AD susceptibility was observed in IGAP and confirmed in a follow-up study (Lambert et al., 2013b; Ruiz et al., 2014). Additionally, *FERMT2* rs17125924 was associated with increased AD risk (Kunkle et al., 2019). *FERMT2*, also known as *KIND2*, is a gene encoding proteins from kindlin family (Lai-Cheong et al., 2010). Kindlin-2 is an integrin-interacting protein, mediating activation of integrin and cell-extracellular matrix interactions (Lai-Cheong et al., 2010; Wei et al., 2014). Carrasquillo et al. showed strong association of *PCDH11X* rs5984894 with LOAD susceptibility (Carrasquillo et al., 2009). *PCDH11X* belongs to the protocadherin gene subfamily of the cadherin superfamily of cell surface receptor molecules. The cadherins mediate cell-cell adhesion and play a role in cell signaling that is critical in the development of the central nervous system (CNS). Rs1466662 within this *DCHS2* was associated with AD (Kamboh et al., 2012a). Protocadherin-23 is another protein from the cadherin superfamily that is expressed in the cerebral cortex and is encoded by *DCHS2* (Höng et al., 2004).

*SHE* rs4474240 was associated with increased LOAD risk (Haddick et al., 2017). Src homology 2 (SH2) domains in SHE are phosphotyrosine binding motifs important in protein-protein interactions in various signaling pathways (Oda et al., 1997). *SORCS1* was identified as potential AD risk locus, but rs2245123 did not show genome-wide significant association (Laumet et al., 2010). SORCS1 is involved in insulin signaling and APP processing (Olgiati et al., 2011). The association of rs7225151 in *SCIMP* region with increased risk for AD was proposed (Moreno-Grau et al., 2019). Another *SCIMP* polymorphism, rs113260531, was recently associated with AD (Jansen et al., 2019). A Src-kinase family mediator SCIMP is palmitoylated transmembrane adaptor, important for immune cell signaling (Draber et al., 2011). *PTPRG* rs7609954 was associated with AD risk (Herold et al., 2016). A member of heterogeneous protein tyrosine phosphatase (PTP) family, PTPRG is a type γ receptor, involved in cell growth, differentiation, mitotic cycle and other processes (Tonks, 2006; Herold et al., 2016). Two novel risk variants for AD were reported for *ABI3* rs616338 and rs28394864 (Sims et al., 2017; Jansen et al., 2019). ABI3 belongs to ABL-interactor (ABI) family proteins, binding partners for the *ABL* kinases (Moraes et al., 2017). Their activation induces cell growth, transformation, and cytoskeletal organization (Satoh et al., 2017). Rs802571 in *CNTNAP2* region was proposed as a novel AD-related protective locus (Hirano et al., 2015). Furthermore, rs114360492 was associated with AD (Jansen et al., 2019). *CNTNAP2* encodes a contactin-associated protein-like 2 transmembrane neurexin, functioning as cell adhesion molecules and receptors in nervous system (Hirano et al., 2015; Saint-Martin et al., 2018). *GOLM1* rs10868366 and rs7019241 showed decreased risk for AD (Li et al., 2008b). GOLM1 is a type II Golgi membrane glycoprotein (Wei et al., 2008). The potential function of GOLM1 in Ras signaling in adenocarcinoma has been recently proposed (Duan et al., 2018). *OR4S1* rs1483121 was associated with AD risk (Zhu et al., 2019). OR4S1 is one of olfactory receptor proteins, G-protein-coupled receptors which represent the largest gene family in the human genome (Milardi et al., 2018). *NYAP1* rs12539172 was associated with decreased LOAD risk (Kunkle et al., 2019). Neuronal tyrosine-phosphorylated phosphoinositide-3-kinase adapter 1 (NYAP1) is involved in the activation of PI3K and the recruitment of the nearby WAVE complex, that regulates brain size and neurite outgrowth in mice (Yokoyama et al., 2011). *TSPOAP1-AS1* rs2632516 and rs2526378 were associated with decreased AD risk (Jun et al., 2017; Witoelar et al., 2018). Benzodiazapine receptor associated protein 1 (TSPOAP1), is another adaptor molecule interacting with Ca^2+^ channels to regulate synaptic transmission (Wang et al., 2000b).

*TREML2* rs9381040 was associated with AD (Marioni et al., 2018). In contrast to TREM2, TREML2 does not interact with DAP12 (Zheng et al., 2016), but it is important in inflammation as a single-pass type I membrane protein expressing an Ig-like V-type domain (Klesney-Tait et al., 2006). *HLA-DRB1* rs9271058 was identified as risk factor (Kunkle et al., 2019). Rs9271192 near *HLA-DRB1* was associated with increased AD risk in IGAP, while intergenic rs6931277 was associated with AD progression (Lambert et al., 2013b; Jansen et al., 2019). HLA-DRB1/HLA-DRB5 locus within the major histocompatibility complex is responsible for numerous immune responses (Rosenthal and Kamboh, 2014). *NECTIN2* rs6859 was associated with LOAD (Abraham et al., 2008; Harold et al., 2009). *NECTIN2* rs6857 and rs41289512 were also associated with AD (Seshadri et al., 2010; Jansen et al., 2019). *NECTIN2* (also known as *PVRL2*) is a gene, encoding for poliovirus receptor 2, immunoglobulin expressed in neuronal cell tissues, that is important in T-cell activation (Whelan et al., 2019).

Two other proteins are important in regulation of cellular processes. A novel *MADD* loci, rs10501320, was recently associated with AD and fasting glucose (Zhu et al., 2019). MADD may play a role in regulating cell proliferation, survival and death through alternative mRNA splicing (Efimova et al., 2004). *PDS5B* rs192470679 was associated with MCI to AD conversion (Lee et al., 2017). PDS5B is part of the cohesin complex, involved in transcriptional regulation, chromosomal compaction and sister chromatid cohesion (Blind, 2020). Stimulating the release of cohesion from chromosomes, PDS5B is considered negative regulator of cohesin DNA-binding function (Carretero et al., 2013; Blind, 2020).

### Biological Regulation

Regulation is a common feature of all living organisms, however complexity of biological regulation is in domain of evolutionary progress. Biological regulation can be addressed as a network of functional relationships, allowing organism to modulate response to changes in internal and external conditions (Bich et al., 2016). Genes and key SNPs, implemented in biological regulation, associated with AD risk in GWAS and meta-analyses, are summarized in Table 3.

**Table 3:** Genes in biological regulation influencing the risk for AD.

**Gene locus**	**SNP**	**Significance for AD risk**	**OR (95 % CI)**	**Study**
*ACE*	rs4293 G>A rs138190086 G>A	0.014 7.5 × 10^−9^	1.22 (1.04–1.41) 1.32 (1.20–1.45)	Webster et al., 2010 Kunkle et al., 2019
*CASS4*	rs7274581 T>C rs6014724 A>G rs6024870 G>A	2.5 × 10^−8^ 6.56 × 10^−10^ 3.5 × 10^−8^	0.88 (0.84–0.92) 0.88 (0.85–0.92)	Lambert et al., 2013b Jansen et al., 2019 Kunkle et al., 2019
*CD2AP*	rs9349407 G>C rs10948363 A>G rs9381563 C>T rs9473117 A>C	8.6 × 10^−9^ 5.2 × 10^−11^ 2.52 × 10^−10^ 1.2 × 10^−10^	1.11 (1.07–1.15) 1.12 (1.07–1.18) 1.10 (1.07–1.13) 1.09 (1.06–1.12)	Hollingworth et al., 2011 Naj et al., 2011 Lambert et al., 2013b Jansen et al., 2019 Kunkle et al., 2019
*CD33*	rs3865444 C>A	1.6 × 10^−9^ 1.6 × 10^−9^ 6.34 × 10^−9^	0.91 (0.88–0.93) 0.89 (0.86–0.93)	Hollingworth et al., 2011 Naj et al., 2011 Jansen et al., 2019
*EPHA1*	rs11767557 T>C rs11771145 G>A rs6973770 G>A rs7810606 T>C rs10808026 C>A	3.4 × 10^−4^ 6.0 × 10^−10^ 0.00216 1.70 × 10^−6^ 1.10 × 10^−13^ 0.001 3.59 × 10^−11^ 1.30 × 10^−10^	0.90 (0.86–0.93) 0.87 (0.83–0.91) 0.91 (0.87–0.94) 0.90 (0.88–0.93) 0.70 (0.56–0.87) 0.90 (0.88–0.93)	Hollingworth et al., 2011 Naj et al., 2011 Kamboh et al., 2012b Seshadri et al., 2010 Lambert et al., 2013b Reitz et al., 2013 Jansen et al., 2019 Kunkle et al., 2019
*HBEGF*	rs11168036 T>G	3.2 × 10^−7^	1.12 (1.07–1.17)	Jun et al., 2016
*IL6R*	rs2228145 A>C	3.0 × 10^−4^	1.3 (1.12 – 1.48)	Haddick et al., 2017
*PDCL3*	rs1513625 G>T	4.28 × 10^−8^		Herold et al., 2016
*TNK1*	rs1554948 T>A	6.3 × 10^−5^	1.19 (1.08–1.3)	Grupe et al., 2007
**Manually annotated genes**				
*CLNK*	rs6448453 A>G	1.93 × 10^−9^		Jansen et al., 2019
*GALP*	rs3745833 C>G	5.0 × 10^−5^	1.2 (1.09–1.32)	Grupe et al., 2007
*HESX1*	rs184384746 C>T	1.24 × 10^−8^		Jansen et al., 2019

Several AD related genes are involved in regulation of the immune system. Specific interleukin gene polymorphisms confer greater risk for AD (Du et al., 2000; Grimaldi et al., 2000; Nicoll et al., 2000). *IL6R* rs2228145 was associated with increased AD risk (Haddick et al., 2017). IL6, a multifunctional cytokine is involved in the regulation of acute inflammatory response and modulation of specific immune response (Akira et al., 1993; Papassotiropoulos et al., 2001). It interacts with IL6 receptor (IL6R). In the nervous system, IL6 has a role in neuronal cell growth and differentiation, as well as neuronal degradation. Multiple evidence suggest the importance of IL6 in AD pathogenesis (Breitner et al., 1986; Peter and Walter, 1991; Campbell et al., 1994). Identifying *CD2AP* as a LOAD risk locus, rs9349407 association with AD was observed (Naj et al., 2011). Increased AD risk effect for rs9349407 was later confirmed (Hollingworth et al., 2011). In IGAP the greatest association with increased AD risk was observed for *CD2AP* rs10948363 (Lambert et al., 2013b). Another two *CD2AP* polymorphisms, rs9381563 and rs9473117, were also associated with AD (Jansen et al., 2019; Kunkle et al., 2019). CD2AP has an important part in the immune system as it binds and clusters CD2 to facilitate junction between T-cells and antigen presenting cells (Rosenthal and Kamboh, 2014). The minor allele of *CD33* rs3865444 was proposed as a protective LOAD locus (Naj et al., 2011). This effect was also confirmed in two other studies (Hollingworth et al., 2011; Jansen et al., 2019). CD33 is a member of the sialic-acid-binding immunoglobulin-like lectins family. It acts as an endocytotic receptor, mediating endocytosis through a mechanism independent of clathrin (Hollingworth et al., 2011; Rosenthal and Kamboh, 2014). It also promotes cell-cell interactions that regulates the innate immune system (Crocker et al., 2007; Tanzi, 2012; Jiang et al., 2014). The level of CD33 was found to be increased in the AD brain and it was in positive correlation with amyloid plaque burden and disease severity (Jiang et al., 2014).

*CASS4* rs7274581 and rs6024870 showed protective function (Lambert et al., 2013b; Kunkle et al., 2019). Furthermore, rs6014724 was associated with AD risk (Jansen et al., 2019). As a member of CAS family, CASS4 directly regulates FAK (focal adhesion kinase) (Deneka et al., 2015). CASS4 is also involved in cytoskeletal function and important in APP metabolism (Karch and Goate, 2015). Association with AD risk was reported for *PDCL3* rs1513625 (Herold et al., 2016). *PDCL3* encodes phosphoducin-like 3, potential modulator of heterotrimeric G-proteins and a chaperone for the VEGF receptor, regulating its ubiquitination and degradation (Srinivasan et al., 2013). *TNK1* rs1554948 association with increased LOAD risk was reported (Grupe et al., 2007). TNK1, a non-receptor protein tyrosine kinase is important in intracellular transduction pathways, involved in THFα induced apoptosis and proliferation of cancer cells (Azoitei et al., 2007; Henderson et al., 2011; Seripa et al., 2018). Rs11767557, located in *EPHA1* promoter region was identified as AD protective locus in multiple studies (Hollingworth et al., 2011; Naj et al., 2011; Kamboh et al., 2012b). Similarly, *EPHA1* rs11771145, rs10808026, and rs6973770 were all associated with decreased AD risk (Seshadri et al., 2010; Lambert et al., 2013b; Reitz et al., 2013; Kunkle et al., 2019). Recently, *EPHA1* rs7810606 was also associated with AD (Jansen et al., 2019). EPHA1 is a member of the ephrin receptor subfamily. Ephrins and Eph receptors are membrane bound proteins involved in cell and axon guidance and in synaptic development and plasticity (Rosenberg et al., 2016). EPHA1 is expressed mainly in epithelial tissues where it regulates cell morphology and motility and it may also have a role in apoptosis and inflammation (Hollingworth et al., 2011).

Two genes, associated with AD, have important role as metabolic hormones. *ACE* rs4293 was identified as a risk loci for LOAD (Webster et al., 2010). Another novel polymorphism in this region was discovered recently. *ACE* rs138190086 association with increased LOAD risk was reported (Kunkle et al., 2019). Angiotensin II, a product of *ACE* gene, primarily known as vasoconstrictor, is also involved in a number of neuropathological processes in AD (Kehoe, 2018). *HBEGF* rs11168036 was associated with increased risk for AD (Jun et al., 2016). HBEGF is an important growth factor, involved in several biological processes like smooth muscle cell growth, skeletal muscle myogenesis, gastrointestinal tract mucosa maintenance, embryo implantation, wound healing and injury repair (Davies-Fleischer and Besner, 1998). HBEGF is widely expressed in the CNS, suggesting its important role in nervous system development (Oyagi and Hara, 2012).

Among all AD risk loci, obtained from GWAS and meta-analyses that were not enriched in GO analysis, additional three were manually annotated to biological regulation and are also summarized in Table 3. Rs6448453 near to *CLNK* was recently associated with AD (Jansen et al., 2019). *CLNK* encodes a protein with important immunomodulatory function, involved in positive regulation of immunoreceptor signaling as a SLP-76 family member (Cao et al., 1999). Since *HESX1* rs184384746 was associated with AD risk, its potential role in AD pathology was discussed (Jansen et al., 2019). Homeobox transcription factor, encoded by *HESX1*, was primarily identified in embryonic stem cells (Thomas and Rathjen, 1992). Grupe et al. reported the association of *GALP* rs3745833 with increased risk for LOAD (Grupe et al., 2007). GALP is a neuropeptide, having important role in the central metabolic control of the reproductive axis (Aziz et al., 2014). It is a ligand in G-protein mediated signal transduction in CNS (Robinson et al., 2006).

### Localization

Localization includes different processes, involved in transport of cells, cell organelles or protein complexes as well as their maintenance in specific location. Genes and key SNPs, implemented in localization, associated with AD risk in GWAS and meta-analyses, are presented in Table 4.

**Table 4:** Genes in localization influencing the risk for AD.

**Gene locus**	**SNP**	**Significance for AD risk**	**OR (95 % CI)**	**Study**
*PLCG2*	rs72824905 C>G rs12444183 A>G	5.38 × 10^−10^ 3.15 × 10^−8^	0.68	Sims et al., 2017 Marioni et al., 2018
*SLC24A4*	rs10498633 G>T rs12590654 G>A rs12881735 T>C	5.5 × 10^−9^ 1.99 × 10^−9^ 1.65 × 10^−10^ 7.4 × 10^−9^	0.91 (0.88–0.94) 0.92 (0.83–1.03) 0.92 (0.89–0.94)	Lambert et al., 2013b Ruiz et al., 2014 Jansen et al., 2019 Kunkle et al., 2019
*SORL1*	rs2101756 A>G rs626885 C>G rs7131432 T>A rs11218313 A>G rs11218343 T>C rs3781834 A>G	0.01923 0.03616 0.03869 0.02026 9.7 × 10^−15^ 2.2 × 10^−9^ 2.9 × 10^−12^ 1.09 × 10^−11^ 9.9 × 10^−9^	1.19 (0.87–1.74) 1.19 (0.97–1.42) 1.73 (1.0–3.67) 1.55 (0.76–2.12) 0.77 (0.72–0.82) 0.81 (0.75–0.87) 0.80 (0.75–0.85) 0.78 (0.72–0.85)	Webster et al., 2008 Lambert et al., 2013b Miyashita et al., 2013 Kunkle et al., 2019 Jansen et al., 2019 Miyashita et al., 2013
*VPS13C*	rs1981916 T>C	0.0016		Meda et al., 2012
**Manually annotated genes**				
*EXOC3L2*	rs597668 T>C	6.5 × 10^−9^	1.17 (1.11–1.23)	Seshadri et al., 2010
*SLC2A9*	rs6834555 G>A	3.0 × 10^−7^	1.40	Hollingworth et al., 2012
*TOMM40*	rs157580 G>A rs157581 T>C rs8106922 A>G rs2075650 A>G rs157582 C>T rs10119 G>A	3.87 × 10^−11^ 9.6 × 10^−54^ <10^−40^ <10^−8^ 3.96 × 10^−14^ 5.4 × 10^−39^ 1.17 × 10^−25^ <1.7 × 10^−8^ 1.8 × 10^−157^ 3.26 × 10^−81^ <10^−40^ <1.7 × 10^−8^ <1.7 × 10^−8^	0.63 (0.59–0.66) 2.73 (2.46–3.05) 0.68 (0.64–0.72) 0.57 2.53 (2.41–2.66) 2.53 (2.37–2.71)	Abraham et al., 2008 Harold et al., 2009 Feulner et al., 2010 Grupe et al., 2007 Abraham et al., 2008 Harold et al., 2009 Pérez-Palma et al., 2014 Seshadri et al., 2010 Harold et al., 2009 Wijsman et al., 2011 Feulner et al., 2010 Seshadri et al., 2010

Four SNPs in *SORL1* (rs2101756, rs11218313, rs626885, and rs7131432) were identified as novel AD related risk alleles (Webster et al., 2008). Twenty-five SNPs in *SORL1* were identified and although none of them showed genome-wide significance for association with AD, rs2070045 was the best predictor of AD risk among them (Laumet et al., 2010). IGAP showed a protective association of *SORL1* rs11218343 with AD (Lambert et al., 2013b). This association was confirmed in two other studies (Jansen et al., 2019; Kunkle et al., 2019). Another two *SORL1* SNPs, rs3781834, and rs11218343 were also associated with LOAD risk (Miyashita et al., 2013). Sorting mechanisms that cause the APP and the β-secretases and γ-secretases to colocalize in the same compartment play an important role in the regulation of Aβ production in AD. APP trafficking is regulated by sortilin related receptors, including SORL1, which binds the APP in the Golgi and reduce availability of precursors for transport, cleavage and transformation in Aβ (Andersen et al., 2005). Decreased expression of *SORL1* leads to overproduction of Aβ (Reitz et al., 2011).

Association of rs1981916 with LOAD could propose *VPS13C* as novel risk locus (Meda et al., 2012). VPS13C potential involvement in cargo selection and sorting into vesicles is consistent with its relocation from mitochondria to cytosol in response to damage. *VPS13C* mutations have been linked to parkinsonism, but the importance of this gene in AD has also been addressed (Lesage et al., 2016).

*PLCG2* rs72824905 was associated with protection against LOAD (Sims et al., 2017). Furthermore, *PLCG2* rs12444183 was associated with AD (Marioni et al., 2018). The product of *PLCG2* gene is involved in the transmembrane transduction of immune signals, that determine the function of various immune cell types in CNS (Patterson et al., 2002; Kim et al., 2015). IGAP identified a protective association with AD risk in *SLC24A4* rs10498633 (Lambert et al., 2013b). This association was later confirmed (Ruiz et al., 2014). Additionally, two studies identified *SLC24A4* rs12590654 and rs12881735 as AD protective loci (Jansen et al., 2019; Kunkle et al., 2019). *SLC24A4* is encoding NCKX4, a sodium-potassium-calcium channel, expressed in human brain (Li et al., 2002). Potential role of *SLC24A4* in nervous system has been discussed, since this gene was linked to neurogenesis pathways (Larsson et al., 2011).

Another three genes, not represented in GO enrichment analysis, were manually annotated to localization and are summarized in Table 4. *TOMM40* is important AD risk locus. *TOMM40* rs157581 was associated with increased AD risk (Grupe et al., 2007). Two other *TOMM40* polymorphisms, rs157580, and rs8106922 were associated with LOAD (Abraham et al., 2008). Both SNPs conferred decreased AD risk in several studies (Harold et al., 2009; Feulner et al., 2010; Pérez-Palma et al., 2014). On the other hand, associations with increased risk for AD were reported for *TOMM40* rs2075650, rs157582, and rs10119 (Seshadri et al., 2010). Numerous studies confirmed the association for rs2075650 (Harold et al., 2009; Feulner et al., 2010; Wijsman et al., 2011). The outer mitochondrial membrane translocase pore subunit (*TOMM40*) forms one of the primary pores *via* which proteins can readily enter the mitochondria. The *TOMM40* gene is the only gene identified in genetic studies to date that presumably contributes to LOAD-related mitochondria dysfunction (Gottschalk et al., 2014). It is encoded on chromosome 19, adjacent to *APOE* region. TOMM40 also impacts brain areas vulnerable in AD, by downstream apoptotic processes that forego extracellular Aβ aggregation. By entering and obstructing the TOMM40 pore, APP induces mechanisms for mitochondrial dysfunction (Devi et al., 2006). As *APOE* and *TOMM40* genomic regions are in close proximity, their potentially interacting effect in mitochondrial function in AD progression is discussed (Roses et al., 2010). Seshadri et al. reported an association between *EXOC3L2* rs597668 and increased LOAD risk (Seshadri et al., 2010). EXOC3L2 (exocyst complex component 3-like 2) is involved in vesicle targeting during exocytosis of proteins and lipids. *SLC2A9* rs6834555 was associated with increased LOAD risk (Hollingworth et al., 2012). The *SLC2A9* encodes for GLUT9, urate transporter, that was initially characterized as a glucose transporter (Vitart et al., 2008; Ebert et al., 2017).

### Genes With No Known GO Function

The remaining 13 genes could not be associated with any of the four main enriched pathways, even though they were linked to risk for AD in GWAS and meta-analysis. Although some of them were linked to a specific function in the literature, they have not been annotated with any of the GO terms. Genes and key SNPs with no known function associated with AD risk in GWAS and meta-analyses are summarized in Table 5.

**Table 5:** Genes with no known GO function influencing the risk for AD.

**Gene locus**	**SNP**	**Significance for AD risk**	**OR (95 % CI)**	**Study**
*ARL17B*	rs2732703 T>G	5.8 × 10^−9^	0.73 (0.65–0.81)	Jun et al., 2016
*BTBD16*	rs10510109 G>T	0.0015		Wang et al., 2017
*CDR2L*	rs71380849 C>A	9.1 × 10^−7^	1.47 (1.26–1.71)	Jun et al., 2016
*IGHV1-68*	rs79452530 C>T	2.36 × 10^−8^	0.89	Witoelar et al., 2018
*KRBOX4*	rs7876304 T>C	9.0 × 10^−4^		Meda et al., 2012
*LHFPL6*	rs9315702 C>A	1.52 × 10^−8^		Melville et al., 2012
*MBLAC1*	rs35991721 G>T	1.44 × 10^−9^	0.92 (0.90–0.95)	Broce et al., 2019
*MS4A4A*	rs4938933 C>T rs10792258 C>T rs2081545 C>A	1.7 × 10^−9^ 0.010 1.55 × 10^−15^	0.88 (0.85–0.92) 0.79 (0.66–0.95)	Naj et al., 2011 Logue et al., 2011 Jansen et al., 2019
*MS4A4E*	rs670139 G>T	1.4 × 10^−9^	1.09 (1.06–1.12)	Hollingworth et al., 2011
*MS4A6A*	rs610932 T>G rs983392 A>G	1.8 × 10^−14^ 6.1 × 10^−16^	0.91 (0.88–0.93) 0.90 (0.87–0.92)	Hollingworth et al., 2011 Lambert et al., 2013b
*NDUFAF6*	rs7812465 T>C	0.0166		Wang et al., 2017
*PPP4R3A*	rs2273647 C>T	3.41 × 10^−15^		Christopher et al., 2017
*ZCWPW1*	rs1476679 C>T rs1859788 A>G	5.6 × 10^−10^ 2.22 × 10^−15^	0.91 (0.89–0.94)	Lambert et al., 2013b Jansen et al., 2019

*BTBD16* rs10510109 was identified as novel AD related polymorphism (Wang et al., 2017). *CDR2L* was previously associated with ovarian cancer and cerebellar degeneration (Raspotnig et al., 2017). *CDR2L* rs71380849 was associated with increased risk for AD (Jun et al., 2016). Increased risk for AD was reported for *MBLAC1* rs35991721 (Broce et al., 2019). Binding with metals is a major function of MBLAC1, encoding for metallo-β-lactamase domain-containing protein in the brain (Fagerberg et al., 2014). It is involved in hydrolysis of different substrates and metabolic intermediates (Gibson et al., 2018). Electron transport chain in mitochondria enables proton motor force generation *via* redox reactions. Genome-wide significant association with T2D and AD was observed for rs7812465 in *NDUFAF6* region (Wang et al., 2017). A protein involved in the assembly of mitochondrial respiratory chain complex I is encoded by *NDUFAF6* (also known as *C8orf38*) (Zurita Rendón and Shoubridge, 2012).

Although the exact function of *IGHV1-68* is not understood to date, rs79452530 in this gene showed decreased risk for AD (Witoelar et al., 2018). *IGHV1-68* is a pseudogene within the immunoglobulin heavy chain (IGH) locus, contributing to the diverse and specific Ig forming in the adaptive immunity (Matsuda et al., 1998). *LHFPL6* rs9315702 was associated with AD-related phenotype of hippocampal volume (Melville et al., 2012). LHFPL tetraspan subfamily member 6 protein, also known as lipoma HMGIC fusion partner is encoded by *LHFPL6* gene with no confirmed function, although a recent study evaluated *LHFPL6* as a bone mass regulator in mice (Mesner et al., 2019).

*ARL17B* rs2732703 was associated with decreased risk of AD in *APOE4* negative population (Jun et al., 2016). ARL17B is localized to the Golgi apparatus and is potentially involved in modulation of vesicle budding and is a known activator of cholera toxin catalytic subunit of ADP-ribosyltransferase (Pasqualato et al., 2002).

*KRBOX4* rs7876304 showed significant association with LOAD (Meda et al., 2012). KRBOX4 is a potential transcriptional regulator with no confirmed function. IGAP revealed a significant protective association of *ZCWPW1* rs1476679 (Lambert et al., 2013b). Recently, rs1859788 in *ZCWPW1* region was also associated with AD (Jansen et al., 2019). Zinc-finger ZCWPW1 is another gene involved in histone modification and epigenetic regulation (He et al., 2010). *PPP4R3A* rs2273647 showed protective effect in risk for AD (Christopher et al., 2017). It encodes a regulatory subunit PPP4R3A of serine/threonine phosphatase (Chowdhury et al., 2008). A novel susceptibility protective locus for AD was *MS4A6A* rs610932, while *MS4A4E* rs670139 showed increased risk for AD (Hollingworth et al., 2011). Tan et al. showed a significant association of *MS4A6A* rs610932 with the risk of LOAD (Tan et al., 2013). In IGAP *MS4A6A* rs983392 had a protective function (Lambert et al., 2013b). *MS4A4A* rs2081545 was associated with AD, while protective function of *MS4A4A* rs4938933 and rs10792258 in AD was also reported (Logue et al., 2011; Naj et al., 2011; Jansen et al., 2019).

## Genes Associated With AD Biomarker Levels

**Figure 3:**
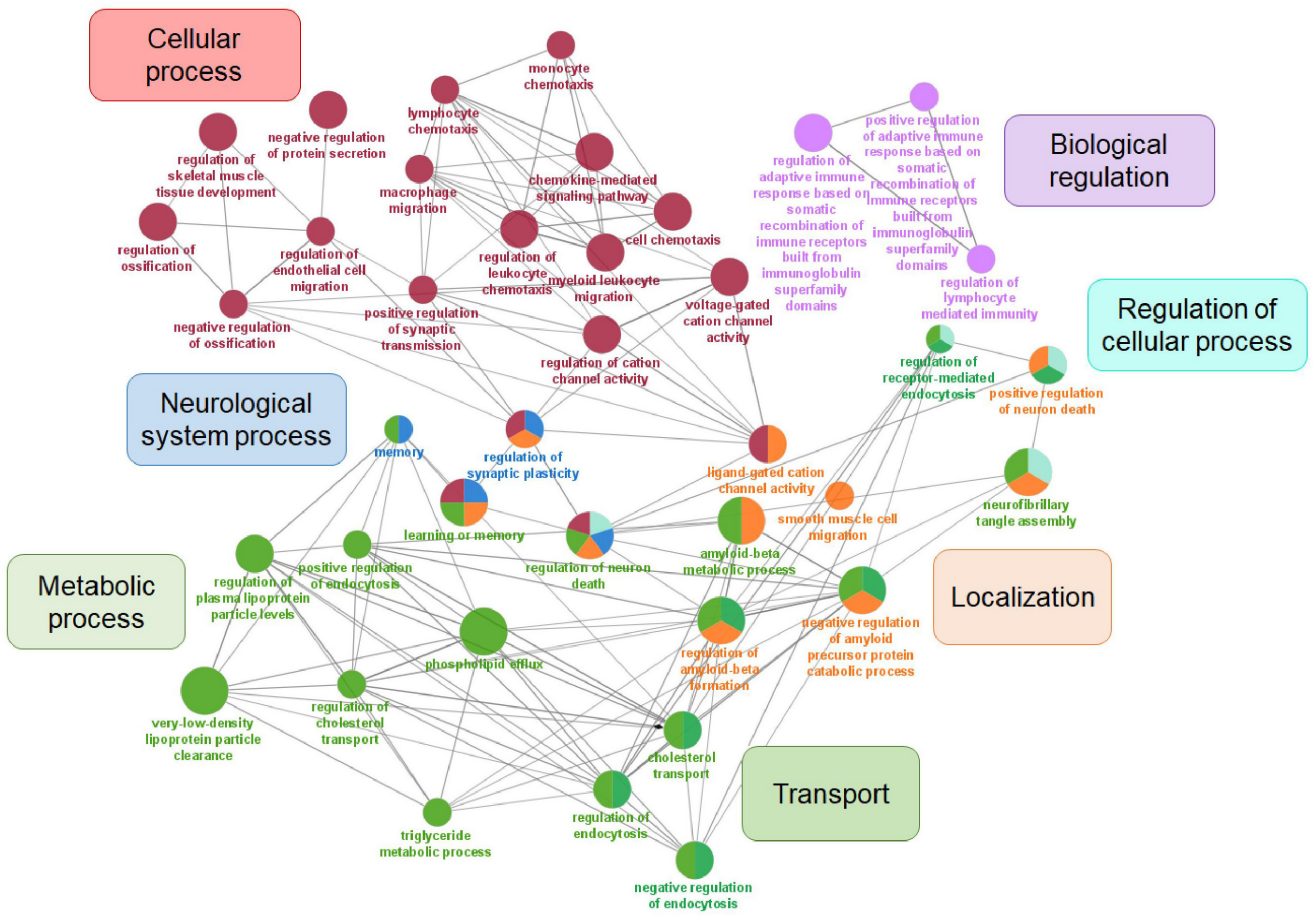
Visualization of GO analysis in AD biomarker gene set. Genes associated with AD biomarkers were stratified according to GO – biological process. They are clustered in seven parental categories and represented with specific color of the node. Biological processes that can be assigned to multiple parental categories, are represented with multiple color-pie chart.

Changes in cerebrospinal fluid (CSF) and blood plasma biomarker levels can predict neurodegenerative changes in AD progression and memory decline and are often used in clinical diagnostics. Except for their diagnostic potential, biomarkers can be applied in studies of AD molecular mechanisms and could be used to monitor the biochemical effects of potential disease intervention (Masters et al., 2015; Efthymiou and Goate, 2017). Genetic variability in different molecular pathways can contribute to differences in biomarker levels. The search for early, reliable and accurate biomarkers for AD progression exceeds genetic approach. Epigenetic factors may play an important role, and the potential of non-coding regulatory RNAs, especially miRNA as biomarkers of AD progression in body fluids has been extensively studied (Takousis et al., 2019). Furthermore, functional neuroimaging provides insight into metabolic and biochemical alterations in the brain, such as glucose metabolism, perfusion, deposition of Aβ and tau protein aggregation (Valotassiou et al., 2018). Thus, GWAS studies and meta-analyses often implement genome-wide genetic data in biomarker studies, to associate mutations or polymorphisms with measurable changes in components of body fluids and brain imaging in AD (Table 6). Apart from identifying novel genetic risk loci, Beecham et al. performed the assessment of the presence of neurofibrillary plaques and tangles, immunohistochemical detection of α-synuclein and neuropathological evaluation in most of the IGAP identified risk loci (Beecham et al., 2014). In the biomarker gene set, seven major GO categories were enriched: cellular process, metabolic process, biological regulation, localization, transport, regulation of cellular process, and neurological system process (Figure 3). Since biomarker gene set enrichment resulted in three additional major categories, some of the genes can be found in different major categories compared to AD risk gene set. Furthermore, transport can be understood as a subcategory of metabolic process, while regulation of cellular process is part of biological regulation and cellular process.

**Table 6:** Genes associated with biomarker levels for AD, according to their GO function.

**Gene locus**	**SNP**	**Biomarker association**	**Significance for biomarker association**	**OR (95 % CI)**	**Study**
**Metabolic process**					
*ABCA7*	rs4147929 A>G	NPs + NFTs	0.01	1.24	Beecham et al., 2014
*APOC1*	rs4420638 A>G rs10414043 G>A rs7256200 G>T rs483082 G>T rs438811 C>T	CSF t-tau/Aß_1−42_ NFTs NFTs NFTs NFTs	1.97 × 10^−14^ 1.64 × 10^−13^ 1.77 × 10^−13^ 1.77 × 10^−11^ 2.25 × 10^−11^		Li et al., 2017 Dumitrescu et al., 2019
*APOE*	rs429358 T>C rs769449 G>A rs7259620 G>A	MRI GM NPs + NFTs NFTs CSF Aβ_1−42_, p-tau, p-tau/Aβ_1−42_, t-tau/Aβ_1−42_ Brain Aβ level (PET) NFTs CSF t-tau/Aß_1−42_ NFTs	<10^−7^ 2.83 × 10^−11^ 2.96 × 10^−31^ <10^−6^ 5.5 × 10^−14^ 2.55 × 10^−14^ 3.62 × 10^−16^ 3.91 × 10^−8^		Shen et al., 2010 Beecham et al., 2014 Dumitrescu et al., 2019 Kim et al., 2011 Ramanan et al., 2014 Dumitrescu et al., 2019 Li et al., 2017 Dumitrescu et al., 2019
*CLU*	rs9331896 C>T	NFTs	0.042	0.85	Beecham et al., 2014
*PICALM*	rs10792832 A>G	NPs + NFTs	1.3 × 10^−4^		Beecham et al., 2014
*VLDLR*	rs7867518 T>C	CSF t-tau	0.00583		Huang et al., 2017a
**Manually annotated genes in metabolic process**					
*EPC2*	rs2121433 T>C rs1374441 G>A rs4499362 C>T rs10171238 C>T	CSF t-tau CSF t-tau CSF t-tau, t-tau/Aβ_1−42_ CSF t-tau, t-tau/Aβ_1−42_	<10^−6^ <10^−6^ <10^−6^ <10^−6^		Kim et al., 2011
*F5*	rs6703865 G>A	MRI HV	1.14 × 10^−9^		Melville et al., 2012
*GCFC2*	rs2298948 T>C	MRI HV	4.89 × 10^−8^		Melville et al., 2012
*GLIS3*	rs514716 C>T	CSF tau CSF p-tau	1.07 × 10^−8^ 3.22 × 10^−9^		Cruchaga et al., 2013
*SUCLG2*	rs62256378 G>A	CSF Aβ_1−42_	2.5 × 10^−12^		Ramirez et al., 2014
**Cellular process**					
*ANK3*	rs10761514 T>C	MRI data	6.14 × 10^−8^		Huang et al., 2019
*CCL4*	rs6808835 G>T	CSF CCL4 level	1.59 × 10^−13^		Kauwe et al., 2014
*CCR2*	rs3092960 G>A	CSF CCL4 level	4.43 × 10^−11^		Kauwe et al., 2014
*CCRL2*	rs6441977 G>A rs11574428 T>A	CSF CCL4 level CSF CCL4 level	7.66 × 10^−11^ 1.48 × 10^−11^		Kauwe et al., 2014
*GLI3*	rs3801203 C>A	Language performance	3.21 × 10^−9^		Deters et al., 2017
*HDAC9*	rs79524815 T>G	NFTs + cerebral amyloid angiopathy	1.1 × 10^−8^		Chung et al., 2018b
*IL6R*	rs61812598 G>A rs4845622 C>A rs4129267 G>A rs2229238 G>A rs2228145 C>A	CSF IL6R level CSF IL6R level CSF IL6R level CSF IL6R level CSF IL6R level CSF IL6R level	5.91 × 10^−63^ 1.36 × 10^−62^ 2.70 × 10^−62^ 2.29 × 10^−14^ 2.70 × 10^−62^ 5.31 × 10^−5^		Kauwe et al., 2014 Kauwe et al., 2014 Haddick et al., 2017
*MEF2C*	rs190982 G>A	NPs + NFTs	0.0011	0.81	Beecham et al., 2014
*MTUS1*	rs55653268 G>T	MRI HV	0.0097		Chung et al., 2018a
*PTK2B*	rs28834970 T>C	Hippocampal sclerosis	0.046	1.13	Beecham et al., 2014
*RBFOX1*	rs12444565 A>C	Glucose metabolism (PET)	6.06 × 10^−8^		Kong et al., 2018
*SERPINB1*	rs316341 G>A	CSF Aβ_1−42_	1.72 × 10^−8^		Deming et al., 2017
**Manually annotated genes in cellular process**					
*ARHGAP24*	rs111882035 A>G	LMT	2.74 × 10^−8^		Chung et al., 2018a
*BCAM*	rs4803758 G>T	CSF p-tau CSF Aβ_1−42_	3.75 × 10^−4^ 3.12 × 10^−5^		Huang et al., 2017a
*BZW2*	rs34331204 A>C	NFTs	2.48 × 10^−8^		Dumitrescu et al., 2019
*CDK5RAP2*	rs10984186 G>A rs4837766 A>T	*CDK5RAP2* gene expression *CDK5RAP2* gene expression	0.008 2.3 × 10^−5^		Miron et al., 2018
*CLUAP1*	rs17794023 C>T	CSF α-synuclein level	9.56 × 10^−9^		Zhong et al., 2019
*IL1RAP*	rs12053868 A>G	Brain Aβ level (PET)	1.38 × 10^−9^		Ramanan et al., 2015
*MMP3*	rs573521 A>G rs645419 A>G rs679620 A>G rs650108 A>G rs948399 T>C	CSF MMP3 level CSF MMP3 level CSF MMP3 level CSF MMP3 level CSF MMP3 level	2.39 × 10^−44^ 3.26 × 10^−44^ 4.93 × 10^−44^ 1.01 × 10^−10^ 4.29 × 10^−11^		Kauwe et al., 2014
*SPI1*	rs1057233 G>A	*SPI1* gene expression	8.24 × 10^−4^		Huang et al., 2017a
**Biological regulation**					
*CD1A*	rs16840041 G>A rs2269714 C>T rs2269715 C>G	Plasma NFL level Plasma NFL level Plasma NFL level	4.50 × 10^−8^ 4.50 × 10^−8^ 4.83 × 10^−8^	1.63 (1.12–2.36)	Wang et al., 2019b
*CR1*	rs6656401 A>G	CP AD	0.004	1.16	Beecham et al., 2014
*NECTIN2*	rs6857 C>T rs12972970 G>A rs34342646 G>A rs12972156 C>G rs283815 A>G	NFTs NFTs CSF tau CSF p-tau NFTs CSF tau CSF p-tau NFTs CSF tau CSF p-tau NFTs	1.38 × 10^−17^ 5.27 × 10^−12^ 8.24 × 10^−16^ 1.28 × 10^−15^ 1.27 × 10^−11^ 5.54 × 10^−16^ 1.17 × 10^−15^ 5.37 × 10^−12^ 3.03 × 10^−15^ 2.31 × 10^−15^ 9.43 × 10^−14^		Dumitrescu et al., 2019 Dumitrescu et al., 2019 Cruchaga et al., 2013 Dumitrescu et al., 2019 Cruchaga et al., 2013 Dumitrescu et al., 2019 Cruchaga et al., 2013 Dumitrescu et al., 2019
**Manually annotated genes in biological regulation**					
*ATP6V1H*	rs1481950 C>A	CSF BACE level	4.88 × 10^−9^		Hu et al., 2018
*CASS4*	rs7274581 T>C	NPs + NFTs	0.0033	0.73	Beecham et al., 2014
*CD33*	rs3865444 C>A	NPs	0.016	0.86	Beecham et al., 2014
**Localization**					
*ACE*	rs4968782 G>A rs4316 C>T rs4343 G>A	CSF ACE level CSF ACE level CSF ACE level	3.94 × 10^−12^ 1.10 × 10^−8^ 3.71 × 10^−8^		Kauwe et al., 2014
*GRIN2B*	rs10845840 T>C	Temporal lobe atrophy	1.26 × 10^−7^	1.273	Stein et al., 2010
*SORL1*	rs11218343 T>C	NFTs	0.0043	0.68	Beecham et al., 2014
**Manually annotated genes in localization**					
*TOMM40*	rs157580 G>A rs2075650 A>G rs157581 T>C rs11556505 C>T rs71352238 T>C rs184017 T>G rs1160985 C>T rs741780 T>C rs1038025 T>C rs760136 A>G rs1038026 A>G rs34404554 C>G rs11556505 C>T rs71352238 T>C	CSF Aβ_1−42_ CSF Aβ_1−42_, p-tau/Aβ_1−_ _42_, t-tau/Aβ_1−42_ CSF tau CSF p-tau NFTs NFTs NFTs NFTs NFTs NFTs NFTs NFTs NFTs NFTs CSF tau CSF p-tau CSF tau CSF p-tau CSF tau CSF p-tau	<10^−6^ <3.1 × 10^−8^ 4.28 × 10^−16^ 5.81 × 10^−16^ 5.28 × 10^−12^ 1.82 × 10^−14^ 4.73 × 10^−12^ 1.20 × 10^−11^ 5.57 × 10^−14^ 2.07 × 10^−8^ 2.11 × 10^−8^ 2.20 × 10^−8^ 2.39 × 10^−8^ 2.45 × 10^−8^ 4.98 × 10^−12^ 3.48 × 10^−16^ 1.33 × 10^−16^ 3.48 × 10^−16^ 1.68 × 10^−16^ 1.78 × 10^−16^ 2.46 × 10^−16^		Kim et al., 2011 Kim et al., 2011 Cruchaga et al., 2013 Dumitrescu et al., 2019 Dumitrescu et al., 2019 Dumitrescu et al., 2019 Cruchaga et al., 2013 Cruchaga et al., 2013 Cruchaga et al., 2013
**Neurological system process**					
*BCHE*	rs509208 G>C	Brain Aβ level (PET)	2.7 × 10^−8^		Ramanan et al., 2014
*MAPT*	rs242557 G>A	Plasma tau	4.85 × 10^−9^		Chen et al., 2017
**Manually annotated genes in neurological system process**					
*ECRG4*	rs34487851 A>G	NPs + NFTs	2.4 × 10^−8^		Chung et al., 2018b
**No known function**					
*CCDC134*	rs7364180 A>G	CSF Aβ_1−42_	<10^−6^		Kim et al., 2011
*FRA10AC1*	rs10509663 A>G rs116953792 G>T	CSF Aβ_1−42_ CSF Aβ_1−42_	1.1 × 10^−9^ 3.5 × 10^−10^		Li et al., 2015
*LUZP2*	rs7943454 T>C	Plasma NFL level	1.39 × 10^−6^		Li et al., 2018
*MS4A6A*	rs983392 A>G rs7232 T>A	NPs CSF TREM2 level	0.036 1.42 × 10^−15^	0.85	Beecham et al., 2014 Hou et al., 2019
*PPP4R3A*	rs2273647 C>T	Glucose metabolism (PET)	4.44 × 10^−8^		Christopher et al., 2017
*ZCWPW1*	rs1476679 C>T	NFTs	0.018	0.86	Beecham et al., 2014
*ZNF804B*	rs73705514 A>C	LMT	2.86 × 10^−9^		Chung et al., 2018a

*Aβ, amyloid β; CP AD, clinico-pathologic features of Alzheimer's disease; CSF, cerebrospinal fluid; GM, gray matter; HV, hippocampal volume; LMT, logical memory test; MRI, magnetic resonance imaging; NFL, neurofilament light; NFT, neurofibrillary tangles; NPs, neuritic plaques; p-tau, phosphorylated tau at threonine 181; PET, positron emission tomography; t-tau, total tau*.

Several biomarker associated loci overlap with risk-related genes (*CASS4, PICALM, SORL1, APOE, CLU, CD33, CR1, MEF2C, NECTIN2, ZCWPW1, ABCA7, MS4A6A*, and *PTK2B*) linked to molecular pathways identified in this review (Table 6).

It is not surprising that *APOE* locus is extensively studied not only as a risk factor, but also in genome-wide analysis of biomarkers. *APOE* rs429358 was associated with neuroimaging-confirmed brain changes (Shen et al., 2010), PET Aβ deposition (Ramanan et al., 2014), CSF Aβ and tau levels (Kim et al., 2011) as well as neuropathologic features (Beecham et al., 2014; Dumitrescu et al., 2019). *APOE* rs769449 was associated with tau/Aβ ratio (Li et al., 2017), while rs769449 and rs7259620 were associated with neuropathologic features (Dumitrescu et al., 2019). Biomarker associations were observed also for *APOC1* rs10414043, rs7256200, rs483082, and rs438811 (Dumitrescu et al., 2019), while *APOC1* rs4420638 was associated with tau/Aβ ratio (Li et al., 2017). *CDK5RAP2* rs10984186 was associated with higher mRNA expression in hippocampus, while rs4837766 showed reduced total mRNA levels (Miron et al., 2018). Also rs157580 and rs2075650 in *TOMM40*, important regulator of energy metabolism were associated with AD biomarkers (Kim et al., 2011; Cruchaga et al., 2013). *TOMM40* rs157581, rs11556505, rs71352238, rs184017, rs1160985, rs741780, rs1038025, rs760136, and rs1038026 were all associated with neuropathologic features of AD (Dumitrescu et al., 2019). Two additional *TOMM40* polymorphisms (rs11556505, rs71352238) were associated with CSF Aβ and tau levels (Cruchaga et al., 2013). Furthermore *TOMM40* rs34404554 was associated with CSF Aβ/tau levels and neuropathologic features respectively (Cruchaga et al., 2013; Dumitrescu et al., 2019). Similarly, SNPs in *NECTIN2* (rs12972970, rs34342646, and rs12972156), important mediator of immune system, were associated with CSF Aβ and tau levels (Cruchaga et al., 2013). All three associations were also linked to neuropathologic features, together with rs6857 and rs283815 (Dumitrescu et al., 2019). Rs61812598, rs4845622, rs2228145, rs4129267, and rs2229238 in the *IL6* receptor (*IL6R*), were associated with LOAD protein expression in CSF [271]. Furthermore, association of rs2228145 with CSF and serum IL6R levels revealed the effect on age of onset in AD [171]. *GLIS3* rs514716 association with CSF Aβ and tau levels was observed (Cruchaga et al., 2013). *SERPINB1* rs316341 and *SPI1* rs1057233 were associated with CSF Aβ levels (Deming et al., 2017; Huang et al., 2017a). The association between decline in brain glucose metabolism and *PPP4R3A* rs2273647 was recently reported (Christopher et al., 2017). Three *ACE* polymorphisms (rs4968782, rs4316, rs4343) were associated with CSF levels of *ACE* gene product (Kauwe et al., 2014). An association with soluble TREM2 was also observed for *MS4A6A* rs7232 (Hou et al., 2019).

Numerous other GWAS studies investigated associations of genetic polymorphisms with biomarker level changes rather than the risk for AD and identified additional 30 genes that were not associated with AD risk (Figure 1).

### Metabolic Processes

Genes and key SNPs, involved in metabolic processes, associated with biomarkers in GWAS and their meta-analyses, are summarized in Table 6.

Rs7867518, adjacent to *VLDLR*, was associated with CSF total tau levels (Huang et al., 2017a). *VLDLR* encodes for a receptor, regulating lipoprotein binding, with high affinity for very low density lipoprotein (VLDL) and APOE-containing lipoproteins (Sakai et al., 1994).

Four other genes associated with biomarker levels, were manually annotated to metabolic process and are also summarized in Table 6. Enzymes in the TCA play a crucial role in energy metabolism, and provide points of interaction between catabolic and anabolic pathways in different cells types, including neuronal tissue. Ramirez et al. found a significant association between *SUCLG2* rs62256378 and CSF Aβ levels in AD subjects (Ramirez et al., 2014). SUCLG2 is a substrate-specific subunit of succinyl CoA ligase, enabling GTP formation in TCA (Johnson et al., 1998). Experiments in cell cultures indicate the absence of SUCLG2 in astrocytes, microglia, and oligodendrocytes, addressing the question of normal TCA function in brain (Dobolyi et al., 2014). Association of *GCFC2* rs2298948 with AD was reported for brain magnetic resonance imaging (Melville et al., 2012). GCFC2 (GC-rich sequence DNA-binding factor 2) is a regulator of pre-mRNA splicing (Yoshimoto et al., 2014). Multiple polymorphism in *EPC2* region were identified (rs2121433, rs1374441, rs4499362, and rs10171238) that were associated with CSF Aβ levels in AD subjects (Kim et al., 2011). Enhancer of polycomb homolog 2, EPC2, is associated with chromatin repressive complex (Whitton et al., 2016). *F5* rs6703865 was associated with brain magnetic resonance imaging with AD (Melville et al., 2012). Specific function of coagulation factor V (F5), a glycoprotein, is important in clot formation (LaBonte, 2014).

### Cellular Processes

Genes and key SNPs, involved in cellular processes, associated with biomarkers in GWAS and their meta-analyses, are summarized in Table 6.

Association with language performance was observed for *GLI3* rs3801203 in AD patients (Deters et al., 2017). GLI3 is a GLI family zinc-finger protein 3 that in presence or absence of Sonic Hedgehog functions as a mediator of the Sonic Hedgehog pathway (Wang et al., 2000a). A novel *HDAC9* polymorphism rs79524815 was recently associated with neuropathologic traits for AD (Chung et al., 2018b). Control of gene expression through chromatin remodeling is a function of histone deacetylase HDAC9 (Sugo et al., 2010). A novel AD-related locus *RBFOX1* rs12444565 in AD driven neurodegeneration was evaluated with fluorodeoxyglucose PET scanning (Kong et al., 2018). Alternative splicing mechanism regulator, important in erythropoiesis, is encoded by RNA binding protein fox-1 homolog 1 (RBFOX1) (Ponthier et al., 2006). *ANK3* rs10761514 was associated with CSF Aβ levels in AD (Huang et al., 2019). *ANK3* encodes for a membrane protein AnkG, important for spectrin-based anchoring of membrane proteins to the cytoskeleton (Kordeli et al., 1995). Kauwe et al. linked *CCL4* rs6808835 with CCL4 protein expression in CSF of LOAD cases (Kauwe et al., 2014). A macrophage inflammatory protein, product of *CCL4* (C-C motif chemokine 4-like) gene, is important enhancer of immune response (Lien et al., 2017). *MTUS1* rs55653268, identified in cognitively normal and MCI subjects, was associated with AD-related changes in hippocampal volume (Chung et al., 2018a). Findings could lead to the understanding of genetic mechanisms for conversion of normal cognitive or mild cognitive impaired individuals to AD patients. Microtubule-associated tumor suppressor 1 gene (*MTUS1*) encodes ATIP3, inhibitor of extracellular signal-regulated kinase 2 (ERK2) and cell proliferation. Similarly, rs3092960 within *CCR2* encoding for CCL2 receptor was also associated with significant levels of CCR2 protein in CSF (Kauwe et al., 2014). Furthermore, two polymorphisms (rs6441977, rs11574428) in C-C chemokine receptor-like 2 gene (*CCRL2*) were associated with CCRL2 protein expression in CSF as well (Kauwe et al., 2014).

Although not enriched in GO analysis, many other genes were manually annotated cellular processes and are also summarized in Table 6. *IL1RAP* rs12053868 was proposed as a marker for PET Aβ deposition in MCI to AD conversion (Ramanan et al., 2015). Interleukin-1 receptor accessory protein (IL1RAP) is essential in cellular response to *IL1* and involved in several other signaling pathways (Cullinan et al., 1998; Dinarello, 2009). Polymorphisms in *MMP3* gene (rs573521, rs645419, rs679620, rs650108, and rs948399) were associated with biomarker levels, with rs573521 being the best predictor of MMP3 protein expression in CSF in LOAD (Kauwe et al., 2014). Matrix metalloproteinase-3 (MMP3) is important in extracellular matrix remodeling (Banik et al., 2015). The Aβ-induced expression of *MMP3*, as well as potential degrading function of extracellular Aβ were observed in astrocytes (Deb and Gottschall, 2002; White et al., 2006). *CLUAP1* rs17794023 was associated with higher CSF α-synuclein levels, suggesting CLUAP1 (*CLU*-associated protein 1) as a novel AD-related locus (Zhong et al., 2019). Rs4803758 near *BCAM* was associated with CSF levels of phosphorylated tau_181_ and Aβ_42_ (Huang et al., 2017a). *BCAM* is a gene encoding Lutheran blood group glycoprotein, an immunoglobulin important in laminin recognition (Parsons et al., 1995). *ARHGAP24* rs111882035 was associated with memory tests outcomes in MCI individuals (Chung et al., 2018a). A Rho GTPase-activating protein 24, encoded by *ARHGAP24* is important in actin cytoskeleton remodeling and specifically suppresses Rac1 and Cdc42 activity (Lavelin and Geiger, 2005). Another SNP associated with neuropathologic features in AD was rs34331204, an intergenic SNP near basic leucine zipper and W2 domain-containing protein 2 (BZW2) (Dumitrescu et al., 2019). The rat homolog, Bdm2, is highly expressed in brain, suggesting the role of the protein in neurodevelopment (Nishinaka et al., 2000).

### Biological Regulation

Genes and key SNPs, involved in biological regulation, associated with biomarkers in GWAS and their meta-analyses, are summarized in Table 6.

Three *CD1A* polymorphisms: rs16840041, rs2269714, and rs2269715 were associated with increased plasma neurofilament light level, a potential protein biomarker for AD (Wang et al., 2019b). CD1A proteins are another important molecules of immune system, regulating glycolipid and lipid antigen presentation of microbial origin or themselves to T-cells (Zajonc et al., 2003; Moody et al., 2004).

Additionally, one biomarker associated gene was manually annotated to biological regulation (Table 6). A polymorphism in the gene encoding for the V1H subunit of vacuolar ATPase, regulating enzyme activity (Marshansky et al., 2014; Colacurcio and Nixon, 2016), *ATP6V1H* rs1481950 was associated with higher CSF BACE activity (Hu et al., 2018).

### Localization

Genes and key SNPs, involved in localization, associated with biomarkers in GWAS and their meta-analyses, are summarized in Table 6.

*GRIN2B* rs10845840 was reported as a risk loci for AD, associated with temporal lobe atrophy (Stein et al., 2010). *GRIN2B* encodes the NR2B subunit of NMDA receptor that mediates a Ca^2+^ dependent synaptic transmission in the CNS (Hu et al., 2016).

### Neurological System Processes

Genes and key SNPs, involved in neurological system process, GO term specific for biomarker gene set, are summarized in Table 6. *MAPT* rs242557 was associated with plasma tau levels (Chen et al., 2017). *MAPT* encodes for tau, the prominent component of NFTs. H2 haplotype is associated with *MAPT* expression and LOAD risk (Allen et al., 2014). A significant association of *BCHE* rs509208 with PET imaging of cortical Aβ in AD subjects was revealed (Ramanan et al., 2014). Butyrylcholinesterase (BCHE) is a serine esterase, involved in organophosphate ester hydrolysis (Amitay and Shurki, 2009). It is important in neurotransmitter activation and enriched in senile plaques of AD brains (Darvesh et al., 2003).

Among all AD related biomarker loci, obtained from GWAS and meta-analyses that were not enriched in GO analysis, additional 18 were manually annotated.

### Genes With No Known GO Function

The remaining seven genes could not be associated with any of the seven main enriched pathways, even though they were linked biomarker changes in AD GWAS and meta-analysis. Although some of them were linked to a specific function in the literature, they have not been annotated with any of the GO terms. Genes and key SNPs with no known function associated with AD biomarkers in GWAS and meta-analyses are summarized in Table 6.

*CCDC134* rs7364180 was associated with CSF Aβ levels in AD subjects (Kim et al., 2011). Coiled-coil domain-containing protein 134, encoded by *CCDC134* is a proliferation promoting molecule, driving cytokine-like activation of CD8^+^ T-cells (Huang et al., 2014). Association with neuropathologic traits of AD in *ECRG4* rs34487851 was observed (Chung et al., 2018b). *ECRG4* encodes a peptide hormone that is involved in NFT formation, age-related senescence of precursor cells in the CNS and activation of microglia and peripheral mononuclear leukocytes (Kujuro et al., 2010; Woo et al., 2010; Podvin et al., 2016). Two polymorphisms (rs10509663, rs116953792) were associated with CSF Aβ levels in AD patients, proposing *FRA10AC1* as a novel risk locus (Li et al., 2015). FRA10AC1 is a protein of unknown function. The polymorphic CGG/CCG repeats in the 5'-UTR of *FRA10AC1* gene are potential cause of folate-sensitive fragile site *FRA10A* expression (Sarafidou et al., 2004). *LUZP2* was also proposed as a novel AD risk locus as rs7943454 was associated with higher plasma neurofilament light levels (Li et al., 2018). A leucine-zipper protein of unknown function, which is normally expressed only in the brain and the spinal cord, is encoded by *LUZP2* gene (Stepanov et al., 2018). Although the function of a zinc finger protein, encoded by *ZNF804B*, is not known yet, a *ZNF804B* rs73705514 was associated with memory tests outcomes in MCI individuals (Chung et al., 2018a).

## Conclusion and Further Perspectives

Alzheimer's disease is the most prevalent neurodegenerative disorder worldwide. A lot of research focuses on the identification of genetic factors that may contribute to the development and progression of the disease. Numerous GWASs and meta-analyses reported different genetic factors associated with AD risk or biomarker levels. A cumulative effect of small but significant contributions of numerous genetic factors can at least in part elucidate the LOAD progression. The pathogenic processes in AD may be influenced on a personalized basis by a combination of variants in key genes and pathways. Apart from serving as a hallmark of the disease, polymorphisms in various genes might help in early diagnostics and prediction of disease progression. Integration of genetic factors and biomarker status may increase the predictive value of diagnostic or prognostic models.

Through the GO analysis we compiled a list of the most enriched pathways, associated with AD pathology. Among four GO parental categories in AD risk gene set, immune response, APP metabolism, cholesterol metabolism, endocytosis and biological regulation on different levels can be exposed as important AD related biological processes. Furthermore, enrichment analysis on smaller AD biomarker gene set pinpointed three additional parental categories. Besides neurodegeneration, numerous research evidence link AD with neuroinflammation, lipid metabolism as well as receptor mediated endocytosis, supporting scientific background of our analysis. Several identified genes were associated with more than one biologic process, represented in various GO categories. The intersection of different biological processes creates a complex interconnected network, suggesting multi-pathway approach in AD genetic background evaluation is needed. Additionally, manual annotation of genes that were not associated with the most significant pathways in GO analysis, could help to elucidate their function in AD pathogenesis.

This comprehensive summary of genetic variants identified by GWAS studies and their meta-analyses can also provide background for identification of novel molecular targets, and the results may be important for development of personalized medicine. However, GWAS and meta-analyses cannot explain the molecular mechanisms of the contribution of a novel susceptibility locus to the overall genetic risk. Therefore, our compiled and annotated results may serve as a basis for the functional studies of pathophysiological mechanisms of risk genes, identified on a genome-wide scale. Furthermore, better characterization of risk genes functions could enable the stratification of AD patients according to the main molecular mechanisms of pathogenesis, supporting development of tailored and personalized treatment of the disease.

## Data Availability Statement

The original contributions presented in the study are included in the article/Supplementary Material, further inquiries can be directed to the corresponding author/s.

## Author Contributions

DV performed literature search and gene enrichment analysis. DV, KG, and VD participated in writing and editing of manuscript. All authors read and approved the final manuscript.

## Conflict of Interest

The authors declare that the research was conducted in the absence of any commercial or financial relationships that could be construed as a potential conflict of interest.

## Supplementary Material

Supplementary Figure 1 PRISMA flow diagram of the literature search.

Supplementary Table 1 Characteristics of genome-wide association studies (GWAS) included in the review.

Supplementary Table 2 Characteristics of meta-analyses included in the review.

Supplementary Table 3 List of enriched GO biological processes in AD risk gene set.

Supplementary Table 4 List of enriched GO biological processes in AD biomarker gene set.

Supplementary Table 5 Raw data of GO enrichment analysis from ClueGO for AD risk gene set.

Supplementary Table 6 Raw data of GO enrichment analysis from ClueGO for AD risk biomarker set.

## Abbreviations

Aβ: amyloid-β
AD: Alzheimer's disease
APP: amyloid precursor protein
EOAD: Early onset Alzheimer's disease
CNS: central nervous system
GO: Gene Ontology
GWAS: genome-wide association study
IGAP: International Genomics of Alzheimer's Project
LOAD: Late onset Alzheimer's disease
MCI: mild cognitive impairment
NFT: neurofibrillary tangles
PET: positron emission tomography
PSEN1: presenilin-1
PSEN2: presenilin-2
T2D: Type 2 diabetes
TCA: tricarboxylic acid cycle.
