# Supplementary material for: Genetic Variability in Molecular Pathways Implicated in Alzheimer's Disease: A Comprehensive Review
Source: Front Aging Neurosci. 2021 Mar 18;13:646901. doi: 10.3389/fnagi.2021.646901 (PMC8012500; doi:10.3389/fnagi.2021.646901)
Supplement: Supplementary Figure 1 — PRISMA flow diagram of the literature search. [file Data_Sheet_1.PDF]

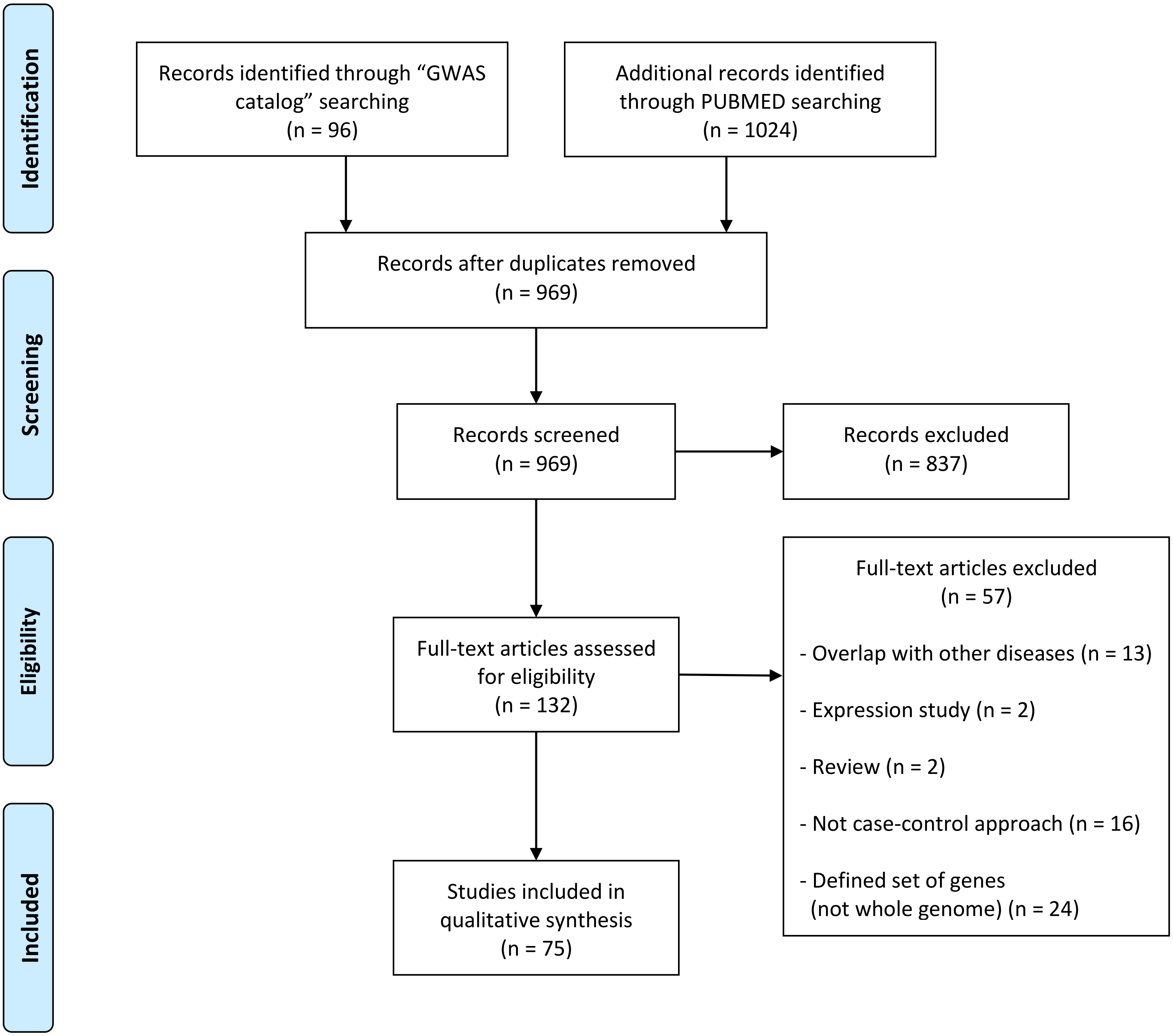
Identification
Screening
Eligibility
Included
Records identified through "GWAS catalog" searching (n = 96)
Additional records identified through PUBMED searching (n = 1024)
Records after duplicates removed (n = 969)
Records screened (n = 969)
Records excluded (n = 837)
Full-text articles assessed for eligibility (n = 132)
Full-text articles excluded (n = 57)
- Overlap with other diseases (n = 13)
- Expression study (n = 2)
- Review (n = 2)
- Not case-control approach (n = 16)
- Defined set of genes (not whole genome) (n = 24)
Studies included in qualitative synthesis (n = 75)
