# Supplementary material for: Genetic Variability in Molecular Pathways Implicated in Alzheimer's Disease: A Comprehensive Review
Source: Front Aging Neurosci. 2021 Mar 18;13:646901. doi: 10.3389/fnagi.2021.646901 (PMC8012500; doi:10.3389/fnagi.2021.646901)
Supplement: Supplementary Table 1 — Characteristics of genome-wide association studies (GWAS) included in the review. [file Table_1.DOCX]

Supplementary Material 1

*Supplementary Table 1: Characteristics of genome-wide association studies (GWAS) included in the review*

| Ethnicity* | Pathology | Initial phase | | Replication phase | | Reference |
| --- | --- | --- | --- | --- | --- | --- |
|  |  | Number of cases** | Number of controls*** | Number of cases** | Number of controls*** |  |
| Caucasian | Mild cognitive impairment, early stage-load | MCI = 367  LOAD = 181 | 209 |  |  | (Meda et al., 2012) |
| Caucasian, European ancestry | Late-onset AD | 844 | 1,255 | 845 | 1,000 | (Carrasquillo et al., 2009) |
| Caucasian, German | Late-onset AD | 491 | 479 |  |  | (Feulner et al., 2010) |
| Caucasian, American and Canadian | AD and mild cognitive impairment | MCI = 163  AD =149 | 151 | MCI = 29 | 402 | (Chen et al., 2017) |
| Spanish | AD, possible or probable diagnosis | 1808 | 2564 |  |  | (Ruiz et al., 2014) |
| Quebec Canadian | Late-onset, sporadic AD | 248 | 91 |  |  | (Miron et al., 2018) |
| African American | Late-onset AD | 1825 | 3784 |  |  | (Mez et al., 2017) |
| Caucasian, American and Canadian | Not stated | MCI = 346  AD =164 | 198 |  |  | (Huang et al., 2019) |
| Caucasian, American and Canadian | MCI to AD converters | MCI = 338  AD = 242 | N.A. |  |  | (Lee et al., 2017) |
| Icelandic population, European, American | AD, population controls, cognitively intact controls | 2261 | 1236 | 2037 | 9727 | (Jonsson et al., 2013) |
| Caucasian, European ancestry | Late-onset AD | 16,097 | 18,077 | 14,041  6,652 | 21,921  8,345 | (Sims et al., 2017) |
| Caucasian, American and Canadian | AD and mild cognitive impairment | MCI = 294  AD = 41 | 160 |  |  | (Ramanan et al., 2015) |
| Caucasian, European ancestry | Late-onset AD | 100 | 102 | 1,145 | 889 | (Haddick et al., 2017) |
| Caucasian, European ancestry | Late-onset AD | 17,008 | 37,154 | 8,572 | 11,312 | (Kauwe et al., 2014) |
| Caucasian, non-Hispanic | Late-onset AD | MCI = 176  AD = 96 | 102 |  |  | (Kim et al., 2011) |
| European, French cohort;  Belgium, Italy, Spain, Finland | Late-onset AD | 2,032 | 5,328 | 3,978 | 3,297 | (Lambert et al., 2009) |
| African American | Late-onset AD | 1968 | 3928 |  |  | (Reitz et al., 2013) |
| African American | Late-onset AD | 513 | 496 |  |  | (Logue et al., 2011) |
| European and USA | AD and cognitively intact controls | 3,941 | 7,848 | 2,023 | 2,340 | (Harold et al., 2009) |
| Caucasian, European ancestry | Neuropathologic AD and controls | 3,887 | 1,027 |  |  | (Beecham et al., 2014) |
| Caucasian, non-Hispanic | AD and mild cognitive impairment | MCI = 330 AD = 160 | 194 |  |  | (Zhong et al., 2019) |
| European ancestry, Spanish | Late-onset AD | 4120 | 3289 | 1943 | 3016 | (Moreno-Grau et al., 2019) |
| Caucasian, European ancestry | AD and mild cognitive impairment | MCI = 581  AD = 190 | 305 |  |  | (Chung et al., 2018a) |
| Caucasian, non-Hispanic | Mild cognitive impairment and cognitive normal controls | MCI = 321 | 224 |  |  | (Wang et al., 2019) |
| European ancestry | AD cases and controls | 1,129 | 2,017 |  |  | (Deming et al., 2017) |
| Caucasian, UK origin | Late onset AD | 1,082 | 1,239 |  |  | (Abraham et al., 2008) |
| European ancestry | AD cases and controls | 1,269 (N.A.) | 1,269 (N.A.) |  |  | (Cruchaga et al., 2013) |
| European ancestry | AD, family based | 1,376 | / | 2,700 | / | (Bertram et al., 2008) |
| Caucasian, non-Hispanic | AD, mild cognitive impairment | MCI = 354  AD = 175 | 204 |  |  | (Shen et al., 2010) |
| Caucasian, UK cohorts  Replication: Swedish cohorts | Cognitive decline | 3,511 |  | 1,367 |  | (Davies et al., 2014) |
| Caucasian, European ancestry | AD, significant memory concern, early and late mild cognitive impairment | SMC = 85  EMCI = 239  LMCI = 207  AD = 113 | 199 |  |  | (Li et al., 2017) |
| Caucasian, non-Hispanic | AD, early and late mild cognitive impairment | EMCI = 190  LMCI = 115  AD = 71 | 179 |  |  | (Ramanan et al., 2014) |
| European ancestry | Late-onset AD | 664 | 422 |  |  | (Coon et al., 2007) |
| European-American sample,  Caribbean, Hispanic in replication | Late-onset AD, family-based | 1,848 | 1,991 | 617 | 573 | (Wijsman et al., 2011) |
| European ancestry, French cohort;  Replication: German cohort | AD cases and controls | 2,259 | 6,017 | 555 | 824 | (Gusareva et al., 2014) |
| Caucasian, non-Hispanic | AD, mild cognitive impairment | MCI = 126  AD = 37 | 59 |  |  | (Kong et al., 2018) |
| Caucasian, UK | Late-onset AD | 753 | 736 | 418 | 249 | (Li et al., 2008) |
| Caucasian, non-Hispanic | AD, significant memory concern, early and late mild cognitive impairment | SMC = 94 EMCI = 283 LMCI = 515 AD = 313 | 370 |  |  | (Deters et al., 2017) |
| European ancestry | AD cases and controls | 3,135 | 463 |  |  | (Chung et al., 2018b) |
| Caribbean Hispanics | Late-onset AD | 2,451 | 2,063 |  |  | (Tosto et al., 2015) |
| Caucasian, European ancestry | AD, mild cognitive impairment | MCI = 335  AD = 79 | 192 | 870 (MCI or AD) | 870 | (Christopher et al., 2017) |
| European ancestry | AD cases and controls | 664 | 422 |  |  | (Webster et al., 2008) |
| Japanese | Late-onset AD | 1,008 | 1,016 | 885 | 985 | (Miyashita et al., 2013) |
| Caucasian, French | AD cases and controls | 2,032 | 5,328 |  |  | (Laumet et al., 2010) |
| Caucasian, UK, USA | Late-onset AD | 1,808 | 2,062 |  |  | (Grupe et al., 2007) |
| European ancestry | AD, mild cognitive impairment | 742 (AD + MCI + CN) | Not stated |  |  | (Stein et al., 2010) |
| Japanese ancestry | Late-onset AD | 816 | 7,992 | 1,011 | 7,212 | (Hirano et al., 2015) |
| European, German | Late-onset AD | 861 | 550 |  |  | (Webster et al., 2010) |
| European, German | AD cases | 363 |  | 515 |  | (Ramirez et al., 2014) |
| Caucasian, non-Hispanic | AD, mild cognitive impairment | MCI = 163  AD = 86 | 91 |  |  | (Hu et al., 2018) |
| Caucasian, non-Hispanic | AD, mild cognitive impairment | MCI = 176  AD = 172 | 179 |  |  | (Li et al., 2018) |
| European | AD, significant memory concern, early and late mild cognitive impairment | EMCI = 254  LMCI = 251  AD = 121 | 215 | SMC = 3  EMCI = 25  LMCI = 61  AD = 62 | 21 | (Li et al., 2015) |
| American, European ancestry | Late-onset AD | 1,291 | 938 |  |  | (Kamboh et al., 2012b) |
| Caucasian, non-Hispanic;  Han Chinese (replication) | AD, mild cognitive impairment | MCI = 350  AD = 96 | 182 | MCI = 130 | 247 | (Hou et al., 2019) |

*as reported by the authors of the respective studies

**if not stated specifically, cases are Alzheimer’s disease patients

***if not stated specifically, controls are cognitive normal adults

AD = Alzheimer's disease; LOAD = Late onset Alzheimer's disease; MCI = Mild cognitive impairment; EMCI = Early mild cognitive impairment; LMCI = Late mild cognitive impairment; SMC = Significant memory concern; CN = cognitive normal

*Supplementary Table 2: Characteristics of meta-analyses included in the review*

| Ethnicity* | Pathology | Initial phase | | Replication phase | | Reference |
| --- | --- | --- | --- | --- | --- | --- |
|  |  | Number of cases** | Number of controls*** | Number of cases** | Number of controls*** |  |
| Caucasian, European ancestry | Late-onset AD | 37,022 | 48,402 |  |  | (Sims et al., 2017) |
| Caucasian, European ancestry | Late-onset AD | 1,145 | 889 |  |  | (Haddick et al., 2017) |
| Caucasian, European ancestry | Late-onset AD | 25,580 | 48,466 |  |  | (Kauwe et al., 2014) |
| European, French cohort;  Belgium, Italy, Spain, Finland | Late-onset AD | 5,887 | 8,508 |  |  | (Lambert et al., 2009) |
| European ancestry, Spanish | Late-onset AD | 11,999 | 9,236 |  |  | (Moreno-Grau et al., 2019) |
| European ancestry, USA, UK, German and Belgium cohort | AD cases and controls | 6,624 | 10,227 |  |  | (Gusareva et al., 2014) |
| Japanese, Korean, Caucasian | Late-onset AD | 12,179 | 12,060 |  |  | (Miyashita et al., 2013) |
| American, European ancestry | Late-onset AD | 2,727 | 3,336 |  |  | (Kamboh et al., 2012b) |
| Caucasian, non-Hispanic  Han Chinese | AD, mild cognitive impairment | MCI = 480  AD = 96 | 329 |  |  | (Hou et al., 2019) |
| Caucasian of multiple ethics (European, American, Hispanic) | Late-onset AD | 2,033 | 14,642 | 1,140 | 1,209 | (Seshadri et al., 2010) |
| Caucasian, European ancestry | Late-onset AD | 17,008 | 37,154 | 8,572 | 11,312 | (Lambert et al., 2013) |
| Caucasian of multiple ethics | Late-onset AD | 8,309 | 7,366 | 3,531  7,650 | 3,565  25,839 | (Naj et al., 2011) |
| Caucasian, European ancestry | Late-onset AD (phase 1)  stratified by parental status (phase 2+3) | 24,087 | 55,058 | 47,793  71,880 | 328,320  383,378 | (Jansen et al., 2019) |
| Caucasian, European ancestry | Late-onset AD | 6,688 | 13,685 | 4,896  8,286 | 4,903  21,258 | (Hollingworth et al., 2011) |
| Caucasian, European ancestry | Late-onset AD, stratified by APOE4 status | APOE4+ 10,352  APOE4- 7,184 | APOE4+ 9,207  APOE4- 26,968 | APOE4+ 1,250  APOE4- 718 | APOE4+ 536  APOE4- 1,699 | (Jun et al., 2016) |
| Caucasian, European ancestry | Neuropathologic AD and controls, stratified by sex and NFT status | 3,887 | 1,027 |  |  | (Dumitrescu et al., 2019) |
| Great Britain | AD stratified by parental status | Maternal AD 27,696  Paternal AD  14,338 | Maternal AD  260,980  Paternal AD  245,941 | 25,580 | 48,466 | (Marioni et al., 2018) |
| Non-Hispanic whites | Late-onset AD | 21,982 | 41,944 | 11,632  35,274 | 18,845  59,163 | (Kunkle et al., 2019) |
| Caucasian, European ancestry | Late-onset AD | 14,406 | 25,849 |  |  | (Huang et al., 2017) |
| Caucasian, European ancestry (IGAP dataset) | Late-onset AD | 17,008 | 37,154 | 10,694 | 14,525 | (Broce et al., 2019) |
| European ancestry | Family-based AD | 2,488 | 979 |  |  | (Herold et al., 2016) |
| European ancestry | AD cases,  GIANT consortium | 6,688  GIANT = 123,685 | 13,685 |  |  | (Hinney et al., 2014) |
| Caucasian  Replication: African American | AD cases, mild cognitive impairment and controls | MCI = 336  AD = 622 | 725 | 188 | 537 | (Melville et al., 2012) |
| European Ancestry, African American, Japanese, Israeli Arab | Late-onset AD | 15,579 | 17,690 | 5,813  21,392 | 20,474  38,164 | (Jun et al., 2017) |
| Caucasian, European ancestry | Late-onset AD,  obesity traits, lipid related traits, sugar related traits | 17,008  OB = 378,993  LP = 262,948  SG = 269,005 | 37,154 |  |  | (Zhu et al., 2019) |
| Caucasian | Late-onset AD | 2,540 | 2,029 |  |  | (Pérez-Palma et al., 2014) |
| Caucasian, African American, Native American | AD cases with and without psychosis | +P = 1,299  -P = 735 | 5,659 |  |  | (Hollingworth et al., 2012) |
| Caucasian, Norway, European ancestry  Replication: Icelandic cohort | Late-onset AD | 28,473 | 55,324 | 5,341 | 110,008 | (Witoelar et al., 2018) |
| European ancestry | Late-onset AD, type 2 diabetes, controls of both population | AD = 17,008  T2D = 12,171 | 37,154 (AD)  56,862 (T2D) |  |  | (Wang et al., 2017) |
| Caucasian American, European | AD cases and controls | 2,222 | Not stated |  |  | (Kamboh et al., 2012a) |

*as reported by the authors of the respective studies

**if not stated specifically, cases are Alzheimer’s disease patients

***if not stated specifically, controls are cognitive normal adults

AD = Alzheimer's disease; MCI = Mild cognitive impairment; T2D = Type 3 diabetes; OB = obesity traits; LP = lipid related traits; SG = sugar related traits; GIANT consortium = Genetic Investigation of Anthropometric Traits (BMI as obesity measurement); +P = AD cases with psychosis; -P = AD cases without psychosis; APOE4+ = APOE4 allele carriers; APOE4- = APOE4 allele non-carriers

*Supplementary Table 3: List of enriched GO biological processes in AD risk gene set*

| GENE LOCUS | BIOLOGICAL PROCESS (GO) |
| --- | --- |
| **Metabolic process** | |
| *ABCA7* | GO:0050435; GO:0030301; GO:0010876; GO:1902992; GO:0034249; GO:0051051; GO:0045807; GO:0065005; GO:1900221; GO:1902003; GO:0032374; GO:0030100; GO:1905952; GO:0060627 |
| *ADAM10* | GO:0050435; GO:0016358; GO:0033619 |
| *APH1B* | GO:0033619 |
| *APOC1* | GO:0030301; GO:0010876; GO:0051051; GO:0065005; GO:0032374; GO:0030100; GO:1905952; GO:0048259; GO:0060627 |
| *APOE* | GO:0050435; GO:0030301; GO:0016358; GO:0010876; GO:0051235; GO:0033619; GO:1902992; GO:0034249; GO:0051051; GO:0045807; GO:0065005; GO:1900221; GO:1902003; GO:0032374; GO:0030100; GO:1905952; GO:0060627 |
| *ATP8B4* | GO:0010876 |
| *BIN1* | GO:0050435; GO:1902992; GO:0034249; GO:0051051; GO:0014015; GO:0065005; GO:1902003; GO:1902959; GO:0030100; GO:0060627 |
| *CELF1* | GO:0034249 |
| *CRY2* | GO:0010876; GO:0051235; GO:0051051; GO:1905952; GO:0010876; |
| *FRMD4A* | GO:0051051; GO:0050714 |
| *OSBPL6* | GO:0030301; GO:0010876; GO:0032374; GO:1905952 |
| *PICALM* | GO:0050435; GO:0016358; GO:1902992; GO:0051051; GO:1902003; GO:1902959; GO:0030100; GO:0048259; GO:0060627 |
| *RAB20* | GO:0030100; GO:0060627 |
| *SLC10A2* | GO:0010876 |
| *SPPL2A* | GO:0033619 |
| *VSNL1* | GO:0051051; GO:0050714; GO:0060627 |
| **Cellular process** | |
| *CLDN18* | GO:0045453; GO:0002521; GO:0002573; GO:0045779; GO:0045637; GO:0046677 |
| *CLU* | GO:0050435; GO:1902656; GO:0071236; GO:0030301; GO:0014009; GO:0002521; GO:0070661; GO:0010876;  GO:0051235; GO:0061518; GO:1902992; GO:0034249; GO:0045807; GO:0050714; GO:1900221; GO:1902003;  GO:0030100; GO:0048259; GO:0060627; GO:0046677; GO:0051208 |
| *COBL* | GO:0016358 |
| *CR1* | GO:0002521 |
| *IL34* | GO:0014009; GO:0002521; GO:0070661; GO:0061518; GO:0030224; GO:0002573; GO:0018108; GO:0014015; GO:0061098; GO:0004713; GO:0045637; GO:0050730 |
| *INPP5D* | GO:0045453; GO:0002521; GO:0070661; GO:0030224; GO:0002573; GO:0045779; GO:0045637 |
| *MEF2C* | GO:0071236; GO:0016358; GO:0002521; GO:0070661; GO:0030224; GO:0002573; GO:0045637; GO:0046677 |
| *MINK1* | GO:0016358 |
| *MS4A2* | GO:1902656; GO:0002521; GO:0070661 |
| *PLEKHA1* | GO:0071236; GO:0046677 |
| *PTK2B* | GO:0045453; GO:0014009; GO:0002521; GO:0051235; GO:0051051; GO:0018108; GO:0004713; GO:0045637; GO:0050730; GO:0046677; GO:0051208 |
| *SERPINB1* | GO:0032691; GO:0051051 |
| *SLC9A7* | GO:0016358 |
| *SPI1* | GO:0071236; GO:0002521; GO:0002573; GO:0045637; GO:0046677 |
| *TP53INP1* | GO:0071236; GO:0046677 |
| *TREM2* | GO:0014009; GO:0002521; GO:0070661; GO:0061518; GO:0002573; GO:0032691; GO:0018108; GO:0045807; GO:0014015; GO:0050714; GO:1900221; GO:0030100; GO:0045637; GO:0050730; GO:0060627 |
| **Biological regulation** | |
| *ACE* | GO:0050435; GO:0070661; GO:0010876; GO:0018108; GO:0061098; GO:0004713; GO:0050730 |
| *CASS4* | GO:0018108; GO:0061098; GO:0004713; GO:0050730 |
| *CD2AP* | GO:0050714; GO:0030100; GO:0048259; GO:0060627 |
| *CD33* | GO:0032691; GO:0051051; GO:0050714 |
| *EPHA1* | GO:0018108; GO:0004713 |
| *HBEGF* | GO:0018108; GO:0061098; GO:0004713; GO:0050730 |
| *IL6R* | GO:0010876; GO:0051235; GO:0045779; GO:0018108; GO:0014015; GO:0050714; GO:1905952; GO:0050730; GO:0046677 |
| *PDCL3* | GO:0018108; GO:0050730 |
| *TNK1* | GO:0018108; GO:0004713 |
| **Localization** | |
| *PLCG2* | GO:0002521; GO:0051235; GO:0045807; GO:0030100; GO:0048259; GO:0060627; GO:0051208 |
| *SLC24A4* | GO:1902656 |
| *SORL1* | GO:0051235; GO:1902992; GO:0034249; GO:0050714; GO:1902003; GO:1902959; GO:0060627 |
| *VPS13C* | GO:0051235 |

*Supplementary Table 4: List of enriched GO biological processes in AD biomarker gene set*

| GENE LOCUS | BIOLOGICAL PROCESS (GO) |
| --- | --- |
| **Metabolic process** | |
| *ABCA7* | GO:0050435; GO:0030301; GO:0007611; GO:0007613; GO:1902992; GO:0045806; GO:0033700; GO:0045807; GO:1902003; GO:0032374; GO:0030100; GO:0097006 |
| *APOC1* | GO:0030301; GO:0045806; GO:0033700; GO:0032374; GO:0030100; GO:0097006; GO:0048259; GO:0006641; GO:0034447 |
| *APOE* | GO:0050435; GO:0030301; GO:0007611; GO:0007613; GO:1902992; GO:0050709; GO:0033700; GO:0045807; GO:0050806; GO:1902003; GO:0032374; GO:0030100; GO:0010594; GO:1901214; GO:0097006; GO:0048167; GO:0006641; GO:0034447 |
| *CLU* | GO:0050435; GO:0060326; GO:0030301; GO:0099094; GO:1902992; GO:1902988; GO:0045807; GO:1901216; GO:1902003; GO:0030100; GO:1901214; GO:0048259 |
| *PICALM* | GO:0050435; GO:0007611; GO:1902992; GO:0045806; GO:1901216; GO:1902003; GO:0030100; GO:1901214; GO:0048259 |
| *VLDLR* | GO:0007611; GO:0007613; GO:0097006; GO:0034447 |
| **Cellular process** | |
| *ANK3* | GO:2001257; GO:0022843 |
| *CCL4* | GO:0060326; GO:0070098; GO:0048247; GO:0002548; GO:0097529; GO:0002688; |
| *CCR2* | GO:0060326; GO:0070098; GO:0048247; GO:0002548; GO:0097529; GO:0002688; GO:0099094; GO:1905517; GO:0002824; GO:0050806; GO:0002822; GO:2001257; GO:0002706; GO:0022843 |
| *CCRL2* | GO:0060326; GO:0070098 |
| *GLI3* | GO:0030278 |
| *HDAC9* | GO:0030279; GO:0050709; GO:0010594; GO:0030278; GO:0048641 |
| *IL6R* | GO:0060326; GO:0002548; GO:0097529; GO:0002688; GO:0030278 |
| *MEF2C* | GO:0007611; GO:0099094; GO:0030279; GO:0002822; GO:2001257; GO:0010594; GO:1901214; GO:0030278; GO:0048641; GO:0048167; GO:0014909; GO:0022843 |
| *MTUS1* | GO:0060326; GO:1905517; GO:0097529; GO:0002688 |
| *PTK2B* | GO:0060326; GO:0070098; GO:0099094; GO:0048247; GO:1905517; GO:0097529; GO:0030279; GO:0050806; GO:0010594; GO:0002688; GO:1901214; GO:0030278; GO:0048167; GO:0022843 |
| *RBFOX1* | GO:0048641 |
| *SERPINB1* | GO:0050709 |
| **Biological regulation** | |
| *CD1A* | GO:0002824; GO:0002822; GO:0002706 |
| *CR1* | GO:0002822; GO:0002706 |
| *NECTIN2* | GO:0002824; GO:0002822; GO:0002706 |
| **Localization** | |
| *ACE* | GO:0050435; GO:0014909 |
| *GRIN2B* | GO:0007611; GO:0099094; GO:1901216; GO:0050806; GO:2001257; GO:1901214; GO:0048167; GO:0022843 |
| *SORL1* | GO:0050435; GO:0007611; GO:1902988; GO:1902003; GO:1901214; GO:0014909; GO:0006641 |
| **Neurological system process** | |
| *BCHE* | GO:0007611 |
| *MAPT* | GO:0007611; GO:0007613; GO:1902988; GO:1901216; GO:1901214; GO:0048167 |

**References**

Abraham, R., Moskvina, V., Sims, R., Hollingworth, P., Morgan, A., Georgieva, L., et al. (2008). A genome-wide association study for late-onset Alzheimer’s disease using DNA pooling. *BMC Med. Genomics* 1. doi:10.1186/1755-8794-1-44.

Beecham, G. W., Hamilton, K., Naj, A. C., Martin, E. R., Huentelman, M., Myers, A. J., et al. (2014). Genome-Wide Association Meta-analysis of Neuropathologic Features of Alzheimer’s Disease and Related Dementias. *PLoS Genet.* 10, e1004606. doi:10.1371/journal.pgen.1004606.

Bertram, L., Lange, C., Mullin, K., Parkinson, M., Hsiao, M., Hogan, M. F., et al. (2008). Genome-wide Association Analysis Reveals Putative Alzheimer’s Disease Susceptibility Loci in Addition to APOE. *Am. J. Hum. Genet.* 83, 623–632. doi:10.1016/j.ajhg.2008.10.008.

Broce, I. J., Tan, C. H., Fan, C. C., Jansen, I., Savage, J. E., Witoelar, A., et al. (2019). Dissecting the genetic relationship between cardiovascular risk factors and Alzheimer’s disease. *Acta Neuropathol.* 137, 209–226. doi:10.1007/s00401-018-1928-6.

Carrasquillo, M. M., Zou, F., Pankratz, V. S., Wilcox, S. L., Ma, L., Walker, L. P., et al. (2009). Genetic variation in PCDH11X is associated with susceptibility to late-onset Alzheimer’s disease. *Nat. Genet.* 41, 192–198. doi:10.1038/ng.305.

Chen, J., Yu, J. T., Wojta, K., Wang, H. F., Zetterberg, H., Blennow, K., et al. (2017). Genome-wide association study identifies MAPT locus influencing human plasma tau levels. *Neurology* 88, 669–676. doi:10.1212/WNL.0000000000003615.

Christopher, L., Napolioni, V., Khan, R. R., Han, S. S., and Greicius, M. D. (2017). A variant in PPP4R3A protects against alzheimer-related metabolic decline. *Ann. Neurol.* 82, 900–911. doi:10.1002/ana.25094.

Chung, J., Wang, X., Maruyama, T., Ma, Y., Zhang, X., Mez, J., et al. (2018a). Genome-wide association study of Alzheimer’s disease endophenotypes at prediagnosis stages. *Alzheimer’s Dement.* 14, 623–633. doi:10.1016/j.jalz.2017.11.006.

Chung, J., Zhang, X., Allen, M., Wang, X., Ma, Y., Beecham, G., et al. (2018b). Genome-wide pleiotropy analysis of neuropathological traits related to Alzheimer’s disease. *Alzheimers. Res. Ther.* 10. doi:10.1186/s13195-018-0349-z.

Coon, K. D., Myers, A. J., Craig, D. W., Webster, J. A., Pearson, J. V., Lince, D. H., et al. (2007). A high-density whole-genome association study reveals that APOE is the major susceptibility gene for sporadic late-onset Alzheimer’s disease. *J. Clin. Psychiatry* 68, 613–618. doi:10.4088/JCP.v68n0419.

Cruchaga, C., Kauwe, J. S. K., Harari, O., Jin, S. C., Cai, Y., Karch, C. M., et al. (2013). GWAS of cerebrospinal fluid tau levels identifies risk variants for Alzheimer’s disease. *Neuron* 78, 256–268. doi:10.1016/j.neuron.2013.02.026.

Davies, G., Harris, S. E., Reynolds, C. A., Payton, A., Knight, H. M., Liewald, D. C., et al. (2014). A genome-wide association study implicates the APOE locus in nonpathological cognitive ageing. *Mol. Psychiatry* 19, 76–87. doi:10.1038/mp.2012.159.

Deming, Y., Li, Z., Kapoor, M., Harari, O., Del-Aguila, J. L., Black, K., et al. (2017). Genome-wide association study identifies four novel loci associated with Alzheimer’s endophenotypes and disease modifiers. *Acta Neuropathol.* 133, 839–856. doi:10.1007/s00401-017-1685-y.

Deters, K. D., Nho, K., Risacher, S. L., Kim, S., Ramanan, V. K., Crane, P. K., et al. (2017). Genome-wide association study of language performance in Alzheimer’s disease. *Brain Lang.* 172, 22–29. doi:10.1016/j.bandl.2017.04.008.

Dumitrescu, L., Barnes, L. L., Thambisetty, M., Beecham, G., Kunkle, B., Bush, W. S., et al. (2019). Sex differences in the genetic predictors of Alzheimer’s pathology. *Brain* 142, 2581–2589. doi:10.1093/brain/awz206.

Feulner, T. M., Laws, S. M., Friedrich, P., Wagenpfeil, S., Wurst, S. H. R., Riehle, C., et al. (2010). Examination of the current top candidate genes for AD in a genome-wide association study. *Mol. Psychiatry* 15, 756–766. doi:10.1038/mp.2008.141.

Grupe, A., Abraham, R., Li, Y., Rowland, C., Hollingworth, P., Morgan, A., et al. (2007). Evidence for novel susceptibility genes for late-onset Alzheimer’s disease from a genome-wide association study of putative functional variants. *Hum. Mol. Genet.* 16, 865–873. doi:10.1093/hmg/ddm031.

Gusareva, E. S., Carrasquillo, M. M., Bellenguez, C., Cuyvers, E., Colon, S., Graff-Radford, N. R., et al. (2014). Genome-wide association interaction analysis for Alzheimer’s disease. *Neurobiol. Aging* 35, 2436–2443. doi:10.1016/j.neurobiolaging.2014.05.014.

Haddick, P. C. G., Larson, J. L., Rathore, N., Bhangale, T. R., Phung, Q. T., Srinivasan, K., et al. (2017). A Common Variant of IL-6R is Associated with Elevated IL-6 Pathway Activity in Alzheimer’s Disease Brains. *J. Alzheimer’s Dis.* 56, 1037–1054. doi:10.3233/JAD-160524.

Harold, D., Abraham, R., Hollingworth, P., Sims, R., Hamshere, M., Pahwa, J. S., et al. (2009). Genome-Wide Association Study Identifies Variants at CLU and PICALM Associated with Alzheimer’s Disease, and Shows Evidence for Additional Susceptibility Genes. *Nat. Genet.* 41, 1088–1093. doi:10.1038/ng.440.Genome-wide.

Herold, C., Hooli, B. V., Mullin, K., Liu, T., Roehr, J. T., Mattheisen, M., et al. (2016). Family-based association analyses of imputed genotypes reveal genome-wide significant association of Alzheimer’s disease with OSBPL6, PTPRG, and PDCL3. *Mol. Psychiatry* 21, 1608–1612. doi:10.1038/mp.2015.218.

Hinney, A., Albayrak, Ö., Antel, J., Volckmar, A. L., Sims, R., Chapman, J., et al. (2014). Genetic variation at the CELF1 (CUGBP, elav-like family member 1 gene) locus is genome-wide associated with Alzheimer’s disease and obesity. *Am. J. Med. Genet. Part B Neuropsychiatr. Genet.* 165, 283–293. doi:10.1002/ajmg.b.32234.

Hirano, A., Ohara, T., Takahashi, A., Aoki, M., Fuyuno, Y., Ashikawa, K., et al. (2015). A genome-wide association study of late-onset Alzheimer’s disease in a Japanese population. *Psychiatr. Genet.* 25, 139–146. doi:10.1097/YPG.0000000000000090.

Hollingworth, P., Harold, D., Sims, R., Gerrish, A., Lambert, J. C., Carrasquillo, M. M., et al. (2011). Common variants at ABCA7, MS4A6A/MS4A4E, EPHA1, CD33 and CD2AP are associated with Alzheimer’s disease. *Nat. Genet.* 43, 429–436. doi:10.1038/ng.803.

Hollingworth, P., Sweet, R., Sims, R., Harold, D., Russo, G., Abraham, R., et al. (2012). Genome-wide association study of Alzheimer’s disease with psychotic symptoms. *Mol. Psychiatry* 17, 1316–1327. doi:10.1038/mp.2011.125.

Hou, X. H., Bi, Y. L., Tan, M. S., Xu, W., Li, J. Q., Shen, X. N., et al. (2019). Genome-wide association study identifies Alzheimer’s risk variant in MS4A6A influencing cerebrospinal fluid sTREM2 levels. *Neurobiol. Aging* 84, 241.e13-241.e20. doi:10.1016/j.neurobiolaging.2019.05.008.

Hu, H., Li, H., Li, J., Yu, J., and Tan, L. (2018). Genome-wide association study identified ATP6V1H locus influencing cerebrospinal fluid BACE activity. *BMC Med. Genet.* 19. doi:10.1186/s12881-018-0603-z.

Huang, M., Deng, C., Yu, Y., Lian, T., Yang, W., and Feng, Q. (2019). Spatial correlations exploitation based on nonlocal voxel-wise GWAS for biomarker detection of AD. *NeuroImage Clin.* 21, 101642. doi:10.1016/j.nicl.2018.101642.

Huang, Y., Zheng, J., Chen, D., Li, F., Wu, W., Huang, X., et al. (2017). Transcriptome profiling identifies a recurrent CRYL1-IFT88 chimeric transcript in hepatocellular carcinoma. *Oncotarget* 8, 40693–40704. doi:10.18632/oncotarget.17244.

Jansen, I. E., Savage, J. E., Watanabe, K., Bryois, J., Williams, D. M., Steinberg, S., et al. (2019). Genome-wide meta-analysis identifies new loci and functional pathways influencing Alzheimer’s disease risk. *Nat. Genet.* 51, 404–413. doi:10.1038/s41588-018-0311-9.

Jonsson, T., Stefansson, H., Steinberg, S., Jonsdottir, I., Jonsson, P. V., Snaedal, J., et al. (2013). Variant of TREM2 associated with the risk of Alzheimer’s disease. *N. Engl. J. Med.* 368, 107–116. doi:10.1056/NEJMoa1211103.

Jun, G., Ibrahim-Verbaas, C. A., Vronskaya, M., Lambert, J. C., Chung, J., Naj, A. C., et al. (2016). A novel Alzheimer disease locus located near the gene encoding tau protein. *Mol. Psychiatry* 21, 108–117. doi:10.1038/mp.2015.23.

Jun, G. R., Chung, J., Logue, M. W., Sherva, R., Farrer, L. A., Mez, J., et al. (2017). Transethnic genome-wide scan identifies novel Alzheimer’s disease loci. *Alzheimer’s Dement.* 13, 727–738. doi:10.1016/j.jalz.2016.12.012.

Kamboh, M. I., Barmada, M. M., Demirci, F. Y., Minster, R. L., Carrasquillo, M. M., Pankratz, V. S., et al. (2012a). Genome-wide association analysis of age-at-onset in Alzheimer’s disease. *Mol. Psychiatry* 17, 1340–1346. doi:10.1038/mp.2011.135.

Kamboh, M. I., Demirci, F. Y., Wang, X., Minster, R. L., Carrasquillo, M. M., Pankratz, V. S., et al. (2012b). Genome-wide association study of Alzheimer’s disease. *Transl. Psychiatry* 2, 1–7. doi:10.1038/tp.2012.45.

Kauwe, J. S. K., Bailey, M. H., Ridge, P. G., Perry, R., Wadsworth, M. E., Hoyt, K. L., et al. (2014). Genome-Wide Association Study of CSF Levels of 59 Alzheimer’s Disease Candidate Proteins: Significant Associations with Proteins Involved in Amyloid Processing and Inflammation. *PLoS Genet.* 10, e1004758. doi:10.1371/journal.pgen.1004758.

Kim, S., Swaminathan, S., Shen, L., Risacher, S. L., Nho, K., Foroud, T., et al. (2011). Genome-wide association study of CSF biomarkers Aβ1-42, t-tau, and p-tau181p in the ADNI cohort. *Neurology* 76, 69–79. doi:10.1212/WNL.0b013e318204a397.

Kong, L.-L., Miao, D., Tan, L., Liu, S.-L., Li, J.-Q., Cao, X.-P., et al. (2018). Genome-wide association study identifies RBFOX1 locus influencing brain glucose metabolism. *Ann. Transl. Med.* 6. doi:10.21037/atm.2018.07.05.

Kunkle, B. W., Grenier-Boley, B., Sims, R., Bis, J. C., Damotte, V., Naj, A. C., et al. (2019). Genetic meta-analysis of diagnosed Alzheimer’s disease identifies new risk loci and implicates Aβ, tau, immunity and lipid processing. *Nat. Genet.* 51, 414–430. doi:10.1038/s41588-019-0358-2.

Lambert, J. C., Heath, S., Even, G., Campion, D., Sleegers, K., Hiltunen, M., et al. (2009). Genome-wide association study identifies variants at CLU and CR1 associated with Alzheimer’s disease. *Nat. Genet.* 41, 1094–1099. doi:10.1038/ng.439.

Lambert, J. C., Ibrahim-Verbaas, C. A., Harold, D., Naj, A. C., Sims, R., Bellenguez, C., et al. (2013). Meta-analysis of 74,046 individuals identifies 11 new susceptibility loci for Alzheimer’s disease. *Nat. Genet.* 45, 1452–1458. doi:10.1038/ng.2802.

Laumet, G., Chouraki, V., Grenier-Boley, B., Legry, V., Heath, S., Zelenika, D., et al. (2010). Systematic analysis of candidate genes for Alzheimer’s disease in a French, genome-wide association study. *J. Alzheimer’s Dis.* 20, 1181–1188. doi:10.3233/JAD-2010-100126.

Lee, E., Giovanello, K. S., Saykin, A. J., Xie, F., Kong, D., Wang, Y., et al. (2017). Single-nucleotide polymorphisms are associated with cognitive decline at Alzheimer’s disease conversion within mild cognitive impairment patients. *Alzheimer’s Dement.* 8, 86–95. doi:10.1016/j.dadm.2017.04.004.

Li, H., Wetten, S., St. Jean, P. L., Upmanyu, R., Surh, L., Hosford, D., et al. (2008). Candidate Single-Nucleotide Polymorphisms From a Genomewide Association Study of Alzheimer Disease. *Arch. Neurol.* 65, 45–53.

Li, J.-Q., Yuan, X.-Z., Li, H.-Y., Cao, X.-P., Yu, J.-T., Tan, L., et al. (2018). Genome-wide association study identifies two loci influencing plasma neurofilament light levels. *BMC Med. Genomics* 11. doi:10.1186/s12920-018-0364-8.

Li, J., Zhang, Q., Chen, F., Meng, X., Liu, W., Chen, D., et al. (2017). Genome-wide association and interaction studies of CSF T-tau/Aβ 42 ratio in ADNI cohort. *Neurobiol. Aging* 57, 247.e1-247.e8. doi:10.1016/j.neurobiolaging.2017.05.007.

Li, Q. S., Parrado, A. R., Samtani, M. N., and Narayan, V. A. (2015). Variations in the FRA10AC1 Fragile Site and 15q21 Are Associated with Cerebrospinal Fluid Aβ1-42 Level. *PLoS One* 10, e0134000. doi:10.1371/journal.pone.0134000.

Logue, M. W., Schu, M., Vardarajan, B. N., Buros, J., Green, R. C., Go, R. C. P., et al. (2011). A comprehensive genetic association study of Alzheimer disease in African Americans. *Arch. Neurol.* 68, 1569–1579. doi:10.1001/archneurol.2011.646.

Marioni, R. E., Harris, S. E., Zhang, Q., McRae, A. F., Hagenaars, S. P., Hill, W. D., et al. (2018). GWAS on family history of Alzheimer’s disease. *Transl. Psychiatry* 8. doi:10.1038/s41398-018-0150-6.

Meda, S. A., Narayanan, B., Liu, J., Perrone-Bizzozero, N. I., Stevens, M. C., Calhoun, V. D., et al. (2012). A large scale multivariate parallel ICA method reveals novel imaging-genetic relationships for Alzheimer’s disease in the ADNI cohort. *Neuroimage* 60, 1608–1621. doi:10.1016/j.neuroimage.2011.12.076.

Melville, S. A., Buros, J., Parrado, A. R., Vardarajan, B., Logue, M. W., Shen, L., et al. (2012). Multiple loci influencing hippocampal degeneration identified by genome scan. *Ann. Neurol.* 72, 65–75. doi:10.1002/ana.23644.

Mez, J., Chung, J., Jun, G., Kriegel, J., Bourlas, A. P., Sherva, R., et al. (2017). Two novel loci, COBL and SLC10A2, for Alzheimer’s disease in African Americans. *Alzheimer’s Dement.* 13, 119–129. doi:10.1016/j.jalz.2016.09.002.

Miron, J., Picard, C., Nilsson, N., Frappier, J., Dea, D., Théroux, L., et al. (2018). CDK5RAP2 gene and tau pathophysiology in late-onset sporadic Alzheimer’s disease. *Alzheimer’s Dement.* 14, 787–796. doi:10.1016/j.jalz.2017.12.004.

Miyashita, A., Koike, A., Jun, G., Wang, L. S., Takahashi, S., Matsubara, E., et al. (2013). SORL1 Is Genetically Associated with Late-Onset Alzheimer’s Disease in Japanese, Koreans and Caucasians. *PLoS One* 8, e58618. doi:10.1371/journal.pone.0058618.

Moreno-Grau, S., de Rojas, I., Hernández, I., Quintela, I., Montrreal, L., Alegret, M., et al. (2019). Genome-wide association analysis of dementia and its clinical endophenotypes reveal novel loci associated with Alzheimer’s disease and three causality networks: The GR@ACE project. *Alzheimer’s Dement.* 15, 1333–1347. doi:10.1016/j.jalz.2019.06.4950.

Naj, A. C., Jun, G., Beecham, G. W., Wang, L., Narayan, B., Buros, J., et al. (2011). Common variants in MS4A4/MS4A6E, CD2uAP, CD33, and EPHA1 are associated with late-onset Alzheimer ’ s disease. *Nat. Genet. Genet.* 43, 436–441. doi:10.1038/ng.801.Common.

Pérez-Palma, E., Bustos, B. I., Villamán, C. F., Alarcón, M. A., Avila, M. E., Ugarte, G. D., et al. (2014). Overrepresentation of glutamate signaling in Alzheimer’s disease: Network-based pathway enrichment using meta-analysis of genome-wide association studies. *PLoS One* 9, e95413. doi:10.1371/journal.pone.0095413.

Ramanan, V. K., Risacher, S. L., Nho, K., Kim, S., Shen, L., McDonald, B. C., et al. (2015). GWAS of longitudinal amyloid accumulation on 18F-florbetapir PET in Alzheimer’s disease implicates microglial activation gene IL1RAP. *Brain* 138, 3076–3088. doi:10.1093/brain/awv231.

Ramanan, V. K., Risacher, S. L., Nho, K., Kim, S., Swaminathan, S., Shen, L., et al. (2014). APOE and BCHE as modulators of cerebral amyloid deposition: A florbetapir PET genome-wide association study. *Mol. Psychiatry* 19, 351–357. doi:10.1038/mp.2013.19.

Ramirez, A., van der Flier, W. M., Herold, C., Ramonet, D., Heilmann, S., Lewczuk, P., et al. (2014). SUCLG2 identified as both a determinator of CSF Aβ1-42 levels and an attenuator of cognitive decline in Alzheimer’s disease. *Hum. Mol. Genet.* 23, 6644–6658. doi:10.1093/hmg/ddu372.

Reitz, C., Jun, G., Naj, A., Rajbhandary, R., Vardarajan, B. N., Wang, L. S., et al. (2013). Variants in the ATP-binding cassette transporter (ABCA7), apolipoprotein e ε4, and the risk of late-onset Alzheimer disease in African Americans. *JAMA - J. Am. Med. Assoc.* 309, 1483–1492. doi:10.1001/jama.2013.2973.

Ruiz, A., Heilmann, S., Becker, T., Hernández, I., Wagner, H., Thelen, M., et al. (2014). Follow-up of loci from the International Genomics of Alzheimer’s Disease Project identifies TRIP4 as a novel susceptibility gene. *Transl. Psychiatry* 4, 2–5. doi:10.1038/tp.2014.2.

Seshadri, S., Fitzpatrick, A. L., Ikram, M. A., DeStefano, A. L., Gudnason, V., Boada, M., et al. (2010). Genome-wide analysis of genetic loci associated with Alzheimer disease. *JAMA - J. Am. Med. Assoc.* 303, 1832–1840. doi:10.1001/jama.2010.574.

Shen, L., Kim, S., Risacher, S. L., Nho, K., Swaminathan, S., West, J. D., et al. (2010). Whole genome association study of brain-wide imaging phenotypes for identifying quantitative trait loci in MCI and AD: A study of the ADNI cohort. *Neuroimage* 53, 1051–1063. doi:10.1016/j.neuroimage.2010.01.042.

Sims, R., Van Der Lee, S. J., Naj, A. C., Bellenguez, C., Badarinarayan, N., Jakobsdottir, J., et al. (2017). Rare coding variants in PLCG2, ABI3, and TREM2 implicate microglial-mediated innate immunity in Alzheimer’s disease. *Nat. Genet.* 49, 1373–1384. doi:10.1038/ng.3916.

Stein, J. L., Hua, X., Morra, J. H., Lee, S., Hibar, D. P., Ho, A. J., et al. (2010). Genome-Wide Analysis Reveals Novel Genes Influencing Temporal Lobe Structure with Relevance to Neurodegeneration in Alzheimer’s Disease. *Neuroimage* 51, 542–554.

Tosto, G., Fu, H., Vardarajan, B. N., Lee, J. H., Cheng, R., Reyes-Dumeyer, D., et al. (2015). F-box/LRR-repeat protein 7 is genetically associated with Alzheimer’s disease. *Ann. Clin. Transl. Neurol.* 2, 810–820. doi:10.1002/acn3.223.

Wang, X. F., Lin, X., Li, D. Y., Zhou, R., Greenbaum, J., Chen, Y. C., et al. (2017). Linking Alzheimer’s disease and type 2 diabetes: Novel shared susceptibility genes detected by cFDR approach. *J. Neurol. Sci.* 380, 262–272. doi:10.1016/j.jns.2017.07.044.

Wang, Z. T., Chen, S. D., Xu, W., Chen, K. L., Wang, H. F., Tan, C. C., et al. (2019). Genome-wide association study identifies CD1A associated with rate of increase in plasma neurofilament light in non-demented elders. *Aging (Albany. NY).* 11, 4521–4535. doi:10.18632/aging.102066.

Webster, J. A., Myers, A. J., Pearson, J. V., Craig, D. W., Hu-Lince, D., Coon, K. D., et al. (2008). Sorl1 as an Alzheimer’s disease predisposition gene? *Neurodegener. Dis.* 5, 60–64. doi:10.1159/000110789.

Webster, J., Reiman, E. M., Zismann, V. L., Joshipura, K. D., Pearson, J. V., Hu-Lince, D., et al. (2010). Whole genome association analysis shows that ACE is a risk factor for Alzheimer’s disease and fails to replicate most candidates from meta-analysis. *Int. J. Mol. Epidemiol. Genet.* 1, 19–30.

Wijsman, E. M., Pankratz, N. D., Choi, Y., Rothstein, J. H., Faber, K. M., Cheng, R., et al. (2011). Genome-wide association of familial late-onset alzheimer’s disease replicates BIN1 and CLU and nominates CUGBP2 in interaction with APOE. *PLoS Genet.* 7, e1001308. doi:10.1371/journal.pgen.1001308.

Witoelar, A., Rongve, A., Almdahl, I. S., Ulstein, I. D., Engvig, A., White, L. R., et al. (2018). Meta-analysis of Alzheimer’s disease on 9,751 samples from Norway and IGAP study identifies four risk loci. *Sci. Rep.* 8, doi:10.1038/s41598-018-36429-6. doi:10.1038/s41598-018-36429-6.

Zhong, X. ling, Li, J. Q., Sun, L., Li, Y. Q., Wang, H. F., Cao, X. P., et al. (2019). A Genome-Wide Association Study of α-Synuclein Levels in Cerebrospinal Fluid. *Neurotox. Res.* 35, 41–48. doi:10.1007/s12640-018-9922-2.

Zhu, Z., Lin, Y., Li, X., Driver, J. A., and Liang, L. (2019). Shared genetic architecture between metabolic traits and Alzheimer’s disease: a large-scale genome-wide cross-trait analysis. *Hum. Genet.* 138, 271–285. doi:10.1007/s00439-019-01988-9.
